# *Clostridium difficile* Toxin CDT Induces Formation of Microtubule-Based Protrusions and Increases Adherence of Bacteria

**DOI:** 10.1371/journal.ppat.1000626

**Published:** 2009-10-16

**Authors:** Carsten Schwan, Bärbel Stecher, Tina Tzivelekidis, Marco van Ham, Manfred Rohde, Wolf-Dietrich Hardt, Jürgen Wehland, Klaus Aktories

**Affiliations:** 1 Institut für Experimentelle und Klinische Pharmakologie und Toxikologie, Albert-Ludwigs-Universität Freiburg, Freiburg, Germany; 2 Fakultät Biologie, Albert-Ludwigs-Universität Freiburg, Freiburg, Germany; 3 Institut für Mikrobiologie, Eidgenössische Technische Hochschule, ETH Zürich, Zürich, Switzerland; 4 Helmholtz-Zentrum für Infektionsforschung, Braunschweig, Germany; University of Illinois, United States of America

## Abstract

*Clostridium difficile* causes antibiotic-associated diarrhea and pseudomembranous colitis by production of the Rho GTPase-glucosylating toxins A and B. Recently emerging hypervirulent *Clostridium difficile* strains additionally produce the binary ADP-ribosyltransferase toxin CDT (*Clostridium difficile* transferase), which ADP-ribosylates actin and inhibits actin polymerization. Thus far, the role of CDT as a virulence factor is not understood. Here we report by using time-lapse- and immunofluorescence microscopy that CDT and other binary actin-ADP-ribosylating toxins, including *Clostridium botulinum* C2 toxin and *Clostridium perfringens* iota toxin, induce redistribution of microtubules and formation of long (up to >150 µm) microtubule-based protrusions at the surface of intestinal epithelial cells. The toxins increase the length of decoration of microtubule plus-ends by EB1/3, CLIP-170 and CLIP-115 proteins and cause redistribution of the capture proteins CLASP2 and ACF7 from microtubules at the cell cortex into the cell interior. The CDT-induced microtubule protrusions form a dense meshwork at the cell surface, which wrap and embed bacterial cells, thereby largely increasing the adherence of *Clostridia*. The study describes a novel type of microtubule structure caused by less efficient microtubule capture and offers a new perspective for the pathogenetic role of CDT and other binary actin-ADP-ribosylating toxins in host–pathogen interactions.

## Author Summary

*Clostridium difficile* is responsible for ∼20 percent of antibiotic-related cases of diarrhea and nearly all cases of pseudomembranous colitis. The pathogens produce two protein toxins (toxins A and B), which inactivate Rho-GTPases of host cells by glucosylation. Recently emerging hypervirulent strains of *C. difficile* release higher amounts of toxins A and B, are resistant towards fluoroquinolones and produce an additional protein toxin called *C. difficile* transferase (CDT). CDT is a binary toxin, which modifies G-actin by ADP-ribosylation, thereby inhibiting actin polymerization. So far the pathogenetic role of CDT is not clear. Here we studied the effects of CDT on human colon carcinoma cells and show that the toxin causes rearrangement of microtubules and formation of long cellular protrusions. The microtubule-based protrusions form a dense meshwork at the cell surface, which wrap and embed *Clostridia*, thereby increasing adherence of the pathogens. We observed similar effects with other members of the family of binary actin-ADP-ribosylating toxins like *C. botulinum* C2 toxin and *C. perfringens* iota toxin. Our findings show a novel type of microtubule structures induced by actin-ADP-ribosylating toxins and propose an important role of these toxins in host–pathogen interactions by their effects on adherence and colonization of *Clostridia*.

## Introduction

Many bacterial protein toxins affect the actin cytoskeleton by modulating the activity of Rho GTPases, including RhoA, Rac and Cdc42 [1]. Prototypic examples of these toxins are *Clostridium difficile* toxins A and B, which cause antibiotic-associated diarrhea and pseudomembranous colitis [2]–[5]. These toxins glucosylate Rho GTPases, thereby inhibiting the signaling functions of Rho proteins [6]. Besides the Rho-glucosylating toxins, up to 35% of *C. difficile* isolates produce an actin-ADP-ribosylating toxin called *C. difficile* transferase (CDT) [7]–[10]. Frequent co-expression of CDT in hypervirulent strains of *C. difficile* (such as Nap1/027) raises the question of its cellular function and pathogenetic role [11].

CDT belongs to the family of binary actin-ADP-ribosylating toxins [12],[13], which are produced by various pathogenic species of the genus *Clostridium* and *Bacillus*, including *C. botulinum* (C2 toxin), *C. perfringens* (iota toxin), *C. spiroforme* (C. spiroforme toxin, CST) and *B. cereus* (vegetative insecticidal proteins, VIP) [1],[14]. These toxins consist of a biologically active, actin-modifying ADP-ribosyltransferase and a separated binding component, which is involved in binding and transport of the enzyme component into the cytosol of target cells.

In the cytosol, the enzymatic component of the toxins ADP-ribosylates G-actin at Arginine-177 [15],[16], thereby blocking actin polymerization. Moreover, ADP-ribosylated actin acts as a capping protein at barbed ends of actin filaments to inhibit elongation and favoring depolymerization of F-actin [17],[18].

Although the effects of ADP-ribosylating toxins on actin are well understood, their role as virulence factors is not clear. Here, we report that the actin-ADP-ribosylating toxins *C. difficile* CDT, *C. perfringens* iota toxin and *C. botulinum* C2 toxin not only affect the actin cytoskeleton but induce the formation of a novel type of microtubule structures, consisting of long microtubule-based protrusions on the surface of epithelial cells, leading to increased adherence of *Clostridia*.

## Results

### Morphological changes of colon epithelial cells induced by actin-ADP-ribosylating toxins

**Figure 1:**
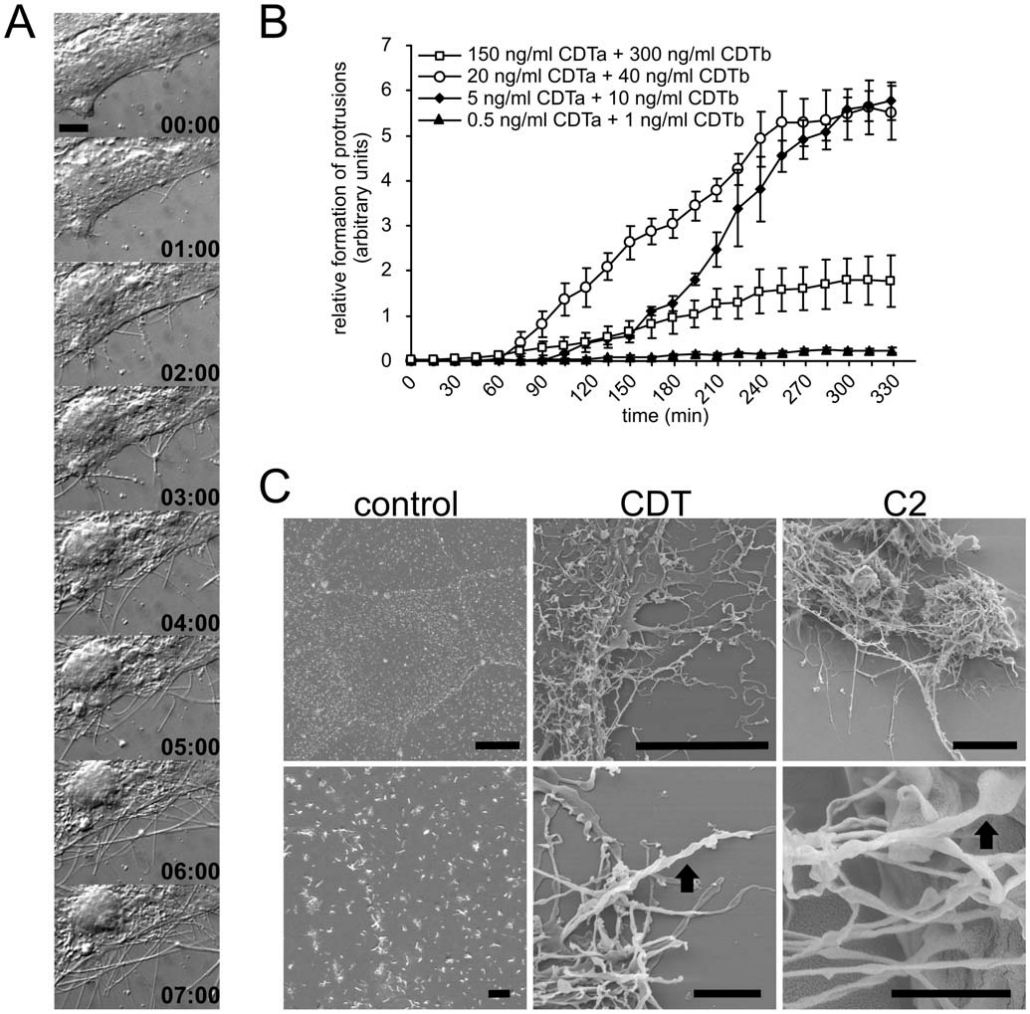
The actin-ADP-ribosylating toxins CDT and C2 toxin induce formation of cellular processes. (A) Subconfluent Caco-2 cells were treated with 20 ng/ml CDTa and 40 ng/ml CDTb. After 1 h the formation of cellular processes starts. Length and number of processes increases over time. In each panel the incubation time (h) is indicated. Scale bar represents 20 µm. (B) Quantification of formation of protrusions. The lengths of all processes of cells were summated every 15 min after addition of the toxins and normalized by the respective section of the cell perimeter. Cells were incubated with the indicated toxin concentrations. 20 ng/ml CDTa +40 ng/ml CDTb and 5 ng/ml CDTa +10 ng/ml CDTb induced the strongest formation of protrusions. Data are given +/−SEM from n≥3 movies and at least 3 cells per movie. (C) Scanning electron microscopy of Caco-2 (left and middle panel) cells treated with 20 ng/ml CDTa and 40 ng/ml CDTb or HT-29 cells (right panel) treated with 250 ng/ml C2I and 500 ng/ml C2II for 2 h. Controls are from Caco-2 cells. Control cells show microvilli at the cell surface. CDT or C2-treated cells show a strong formation of protrusions. Arrow: corkscrew shaped protrusion with leaf-like extension. Scale bar in upper row represents 10 µm. Scale bar in lower row represents 2 µm.

To test the effects of the actin-ADP-ribosylating toxin CDT, we employed the human colon carcinoma cell line Caco-2. Treatment with the toxin caused shrinking and rounding-up of target cells. These morphological changes are typical for all binary actin-ADP-ribosylating toxins and depend on ADP-ribosylation of actin [12],[19]. To get more information on toxin-induced morphological changes, we made use of time-lapse-microscopy (Figure 1A). Surprisingly, we detected toxin-induced formation of cellular processes, occurring earlier than shrinking of the cell body. About 60–90 min after application of CDT (20 ng/ml CDTa and 40 ng/ml CDTb), the binary toxin induced formation of thin cell processes that extended from the surface of epithelial cells (Figure 1A, Video S1). The structures were different in length, usually ranging from 5 - >150 µm with a diameter ranging from 0.05 to 0.5 µm. Neither the enzyme component nor the binding component of CDT alone had any effects on cell morphology and process formation, indicating the specificity of the toxin effect (Figure S1A). Moreover, intoxication with a catalytic inactive mutant of CDTa (E430Q) [20] revealed no formation of protrusions (Figure S1B). On the other hand, identical protrusions were caused by treatment of epithelial cells with other actin-ADP-ribosylating toxins like *C. botulinum* C2 toxin and *C. perfringens iota toxin* (Figure S1C). In addition to Caco-2 cells, formation of toxin-induced protrusions was observed in human colon carcinoma HT-29 cells (see below scanning electron microscopy, Figure 1C, right panel) as well as in primary intestinal epithelial cells isolated from the rat colon (Figure S1D).

Quantification (for method see Figure S2) of toxin-induced formation of protrusions revealed that maximal effects occurred at medium concentrations of CDT (20 ng/ml CDTa and 40 ng/ml CDTb) (Figure 1B). At high concentrations of CDT (150 ng/ml CDTa and 300 ng/ml CDTb) outgrowth of protrusions was rapid and occurred within 40 min but length of protrusions was reduced as compared to medium concentrations. At 5 ng/ml CDTa and 10 ng/ml CDTb, first protrusions were formed after ∼120 min. Similar results were obtained with C2 toxin (data not shown).

Next we employed scanning electron microscopy. These studies showed that CDT-induced formation of protrusions occurred all over the cell surface of Caco-2 cells (Figure 1C). Whereas with time lapse DIC microscopy formation of straight protrusions at rather early time points was observed, scanning electron microscopy at later time points (>3 h) revealed formation of a meshwork of cellular extensions. The protrusions were bended and exhibited contact with each others. Interestingly the shape of protrusions was often twisted in a corkscrew-like manner with small leaf-like extensions (arrows in Figure 1C, lower panel). These structures were clearly distinguishable from short microvilli observed on control cells by their shape and their variation in length (Figure 1C) [21]–[23]. A similar formation of protrusions and a disappearance of microvilli were observed on polarized Caco-2 cells that were grown on filters for 2 weeks to ensure polarization (Figure S3A). In these experiments the cell borders and the presence of tight junctions indicative for polarization were determined by occludin and ZO-2 stainings, respectively (Figure S3B+C).

### Binary toxins affect microtubules

**Figure 2:**
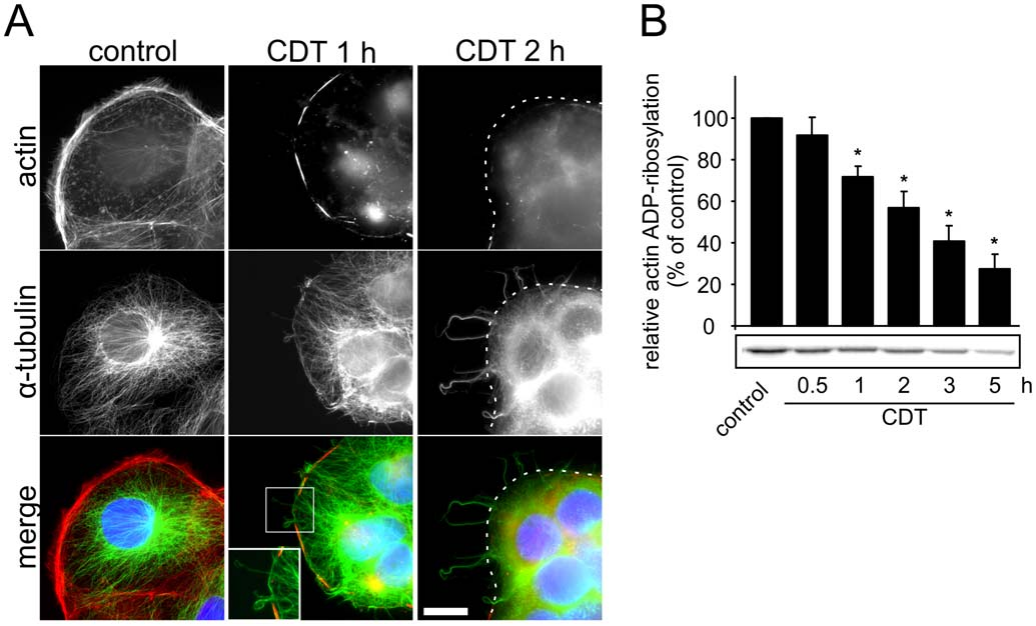
Toxin-induced disruption of the actin cytoskeleton and formation of microtubule-based protrusions. (A) Indirect immunofluorescence of α-tubulin (green) and actin-staining by TRITC-conjugated phalloidin (red) in Caco-2 cells. The nucleus was stained by DAPI (blue). CDT causes increasing disruption of the actin cytoskeleton and a concomitant increased formation of microtubule-based protrusions over time. Cells were treated with 20 ng/ml CDTa and 40 ng/ml CDTb for the indicated times. White dotted line in the right panels indicates cell border. The line was delineated according to a phase contrast picture. White square in lower panel is magnified below. Scale bar represents 20 µm. (B) *In vitro* ^32^P-ADP-ribosylation of actin by the enzyme component C2I after intracellular ADP-ribosylation by CDT. Caco-2 cells were treated with 20 ng/ml CDTa and 40 ng/ml CDTb for the indicated times. Cells were lysed and unmodified actin was subsequently modified by C2I in the presence of [^32^P]NAD. The ^32^P-ADP-ribosylated actin was analyzed by SDS-PAGE and phosphorimaging. C2I-catalyzed ^32^P-ADP-ribosylation of actin in control cells was taken as 100%. Data in columns are given from 3 independent experiments (+/−SEM). Below, the autoradiogram of an ADP-ribosylation from a representative experiment is shown.

Cell surface projections such as filopodia, microspikes or microvilli harbor actin filaments which are thought to be their key structural component [23]. Therefore, we applied TRITC-phalloidin to study whether the toxin-induced protrusions contained F-actin. Whereas in control cells cortical filamentous actin was stained at cell borders (Figure 2A), treatment with CDT (50 ng/ml CDTa and 100 ng/ml CDTb) for 1 h induced redistribution of cortical actin filaments and formation of unstructured F-actin dots. After 2 h, the toxin caused severe destruction of the actin cytoskeleton. However, the protrusions induced by the actin-ADP-ribosylating toxins were not stained by TRITC-phalloidin. By contrast, indirect immunofluorescence with anti-α-tubulin antibodies revealed that the toxin-induced protrusions consisted of microtubules (Figure 2A). One hour after addition of toxin, microtubules started to form bundles, grew along the cell cortex and/or crossed cell borders by forming protrusions at the cell surface. After 2 h of toxin treatment, long bundles of microtubules projected from the cell borders and the intracellular microtubule meshwork was redistributed. Additionally, by confocal microscopy of the cell surface of polarized Caco-2 cells the meshwork of microtubule-based protrusions was clearly visible (Figure S3B+C). A magnification (Figure S3D) of the surface identified the structures seen in scanning electron microscopy (Figure S3A). Altogether, the microscopic studies indicated that the toxins mainly affected the microtubule network at the cell cortex. Accordingly, formation of protrusions was inhibited by addition of nocodazole (not shown).

To study the microtubule-based protrusions in more detail, we used antibodies specific for post-translational modifications of tubulin. In normal growth conditions, Caco-2 cells displayed very low amounts of detyrosinated α-tubulin (Glu-tubulin), which was not increased after disruption of the actin cytoskeleton by toxins (Figure S4A). Also the amount of acetylated tubulin was not enriched after toxin treatment (Figure S4B). As both detyrosinated tubulin and acetylated tubulin are markers for more stabilized microtubules [24],[25], these results indicate that the microtubule-based protrusions mainly consist of dynamic microtubules. Accordingly, antibodies specific for tyrosinated α–tubulin stained microtubules in the protrusions (Figure S4C).

To study whether formation of microtubule protrusions was accompanied and/or preceded by ADP-ribosylation of actin, we analyzed CDT-catalyzed modification of actin in Caco-2 cells in a time course by differential ADP-ribosylation (Figure 2B). To this end, intact cells were treated with CDT for the indicated times. Thereafter, cells were lysed and cellular proteins were incubated with radioactively labeled NAD. ^32^P-ADP-ribosylation of actin was induced by addition of the enzyme component C2I of C2 toxin, which also modifies actin at Arg177. One hour after treatment of cells with CDT, the ^32^P-ADP-ribosylation of actin in the cell lysate was reduced by ∼30%. At this time point the first microtubule extensions were formed at the cell surface, suggesting that formation of protrusions and ADP-ribosylation of actin are concomitant processes.

To address the question, whether the formation of microtubule protrusions was a consequence of actin disruption or an actin independent effect, we tested the microfilament destabilizing drugs cytochalasin D and latrunculin B (Figure S5A) [26]. Both cytochalasin D and latrunculin B induced microtubule protrusions; however, they induced protrusions less efficiently than CDT and rather high concentrations (e.g., 0.5–5 µM latrunculin B or cytochalasin D) were necessary to achieve similar effects on microtubules as CDT. The effects induced by cytochalasin D or latrunculin B started early after addition but the process of outgrowth of protrusions was less pronounced. When CDT was applied 10 min after latrunculin B, the typical strong effect of the ADP-ribosylating toxin on the formation of microtubule protrusions was still observed (data not shown).

Treatment of Caco-2 cells with jasplakinolide, which is known to stabilize F-actin and to enhance polymerization [27], delayed and reduced the formation of microtubule protrusions after treatment with CDT (Figure S5B). By contrast, the Rho GTPase-inactivating toxins *C. difficile* toxin B and *C. limosum* C3 toxin, both of which alter the dynamics of the actin cytoskeleton, did not induce microtubule protrusions (Figure S5C).

### Binary toxins influence microtubule plus end tracking proteins

**Figure 3:**
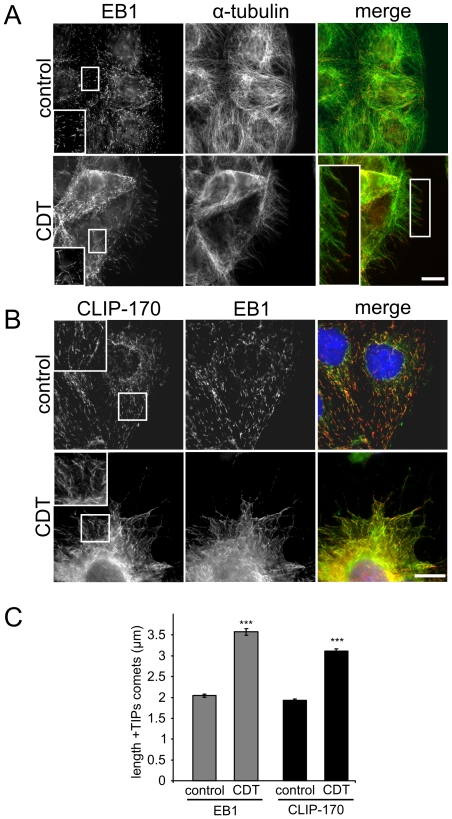
Influence of CDT on EB1 and CLIP-170 at microtubule plus-ends. (A) Indirect immunofluorescence of EB1 (red) and α-tubulin (green) in Caco-2 cells. Control cells show short comet-like EB1 associations at the tip of microtubules. After CDT treatment, EB1 comets are elongated and also present at the tip of microtubule-based protrusions. Cells were treated with 20 ng/ml CDTa and 40 ng/ml CDTb and fixed after 2 h. Areas in white squares show magnifications. Scale bar represents 20 µm. (B) Indirect immunofluorescence of CLIP-170 (green) and EB1 (red) in Caco-2 cells. The nucleus was stained by DAPI (blue). EB1 and CLIP-170 comets are elongated after CDT treatment. Cells were treated with 20 ng/ml CDTa and 40 ng/ml CDTb and fixed after 2 h. Areas in white squares show magnifications. Scale bar represents 20 µm. (C) Quantification of the EB1 and CLIP-170 comet lengths. The figure shows a quantification of the EB1 and CLIP-170 comet length from pictures stained as in Figure 3B. After CDT treatment, both +TIPs have an increased microtubule tip association. +TIPs were measured by Metamorph software. Data are given +/−SEM (*** = p<0.001, n = 100). CDT concentrations were as above.

Our studies showed that CDT affects microtubule formation especially at the cell cortex. Since the dynamics and capturing of microtubules is mainly controlled by plus end-tracking proteins (+TIPs), we studied +TIP end-binding protein 1 (EB1), which is a marker of polymerizing microtubules [28],[29]. Staining of EB1 in control cells revealed a typical pattern with short comet-like structures at the tip of microtubules (Figure 3A) [30]. Measurement of comets yielded a length of ∼2.04+/−0.04 µm. Treatment with CDT increased the length of EB1-decorated tips of microtubules to 3.57+/−0.08 µm (p<0.001) (Figure 3C). Also the ends of most microtubules that crossed cell borders and formed protrusions were decorated with EB1. This indicated that microtubules, which projected from the cell surface into the protrusions, were still in their growth phase. Moreover, these findings suggested that toxin-induced formation of protrusions is at least partially generated by tubulin polymerization and rather not by a mechanism that involves sliding of microtubules. Toxin-induced elongation of EB1 comet length was also observed in HT29 cells and in primary epithelial cells from the rat colon (data not shown).

Similar results as observed for EB1 were obtained for cytoplasmic linker protein 170 (CLIP-170), which is another +TIP member [31] (Figure 3B). The length of microtubule tip decoration with the rescue factor CLIP-170 increased after the treatment with CDT (Figure 3C). Microtubule protrusions, which extended the cell surface, were decorated with both EB1 as well as CLIP-170 after treatment. Additionally, CLIP-115 and EB3 displayed a similar increase in microtubule tip decoration (Figure S6) [32].

**Figure 4:**
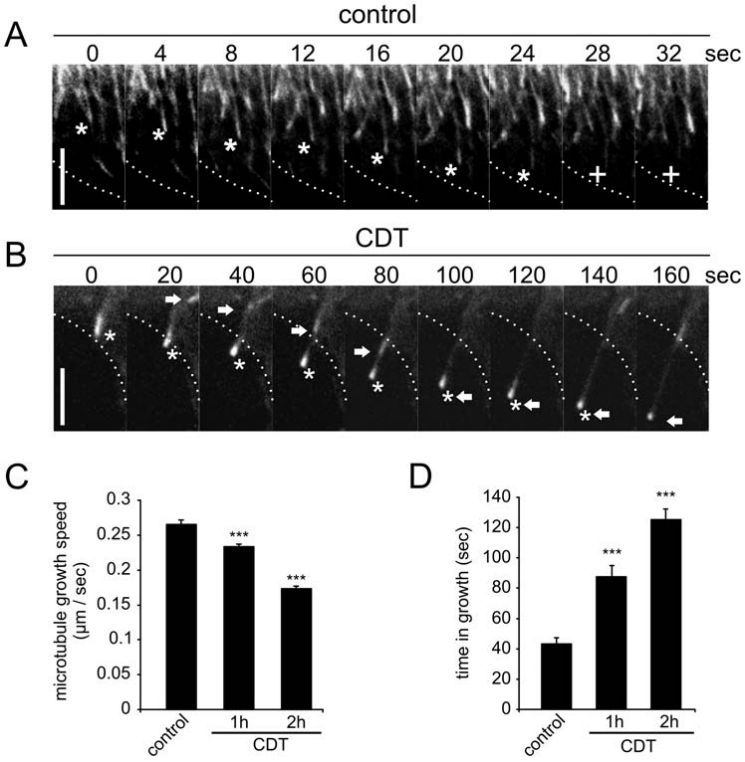
Influence of CDT on microtubule growth. Caco-2 cells transfected with EB3-GFP, were monitored in fluorescence time-lapse microscopy. Cells were recorded 0, 1 and 2 h after treatment with 20 ng/ml CDTa and 40 ng/ml CDTb. (A) Control images of EB3-GFP transfected cells show microtubules losing their EB3-comets as they approach the cell cortex. Asterisk: EB3-GFP comet approaching the cell cortex (white dotted line); cross: same comet during loss of EB3 decoration. (B) Images of CDT-treated (2 h) EB3-GFP transfected cells. Microtubules approaching the cell cortex do not lose EB3-association. Polymerizing microtubule forms initial protrusion and other microtubules follow the same track. Asterisk: EB3-GFP comet forming a protrusion. Arrow: a second EB3-GFP comet following the first into the protrusion. Scale bar in A and B represents 5 µm. Note different time scales in A and B. (C) Quantification of microtubule polymerization rate. After CDT treatment the polymerization rate of microtubules decreases. Growing microtubules (EB3-GFP comets) were tracked over 10 sec at 0 (control), 1 and 2 h after CDT-treatment. Data from 100 comets (5 experiments and 7 different cells) are given +/−SEM (*** = p<0.001). (D) Quantification of microtubule time in growth. After CDT treatment microtubules stay longer in their growth phase. The life time of 30 EB3-GFP comets from appearance to fading was measured (data are given +/−SEM (*** = p<0.001) from ≥5 cells and ≥4 independent experiments).

To analyze the consequences of increased +TIP-association on microtubule dynamics, a GFP-version of the +TIP EB3 was expressed and the cells were analyzed by fluorescence time-lapse microscopy with high temporal resolution (Figure 4A+B, Video S2). Only cells, expressing low levels of the GFP construct, were selected for analysis. The elongation of +TIPs after CDT treatment was also evident in EB3-GFP-transfected cells. The length of GFP-positive comets before and after the treatment with CDT was similar to the endogenous EB1-comets in immunofluorescence. This reflects the similar behavior of the endogenous +TIP as compared to the expressed recombinant EB3-GFP protein. In control cells, +TIP comets faded as soon as they reached the cell cortex. By contrast, in CDT-treated cells the EB-comets crossed the cell cortex and formed initial protrusions. Often microtubules grew along already existing ones as seen by the appearance of EB-comets using the same track into the protrusions. By means of EB3-GFP the rate of polymerization of ∼100 steadily growing microtubules was measured before and after 1 and 2 h of treatment with CDT (Figure 4C). Surprisingly, after the treatment, the rate of polymerization was significantly reduced from 0.26+/−0.006 µm/sec to 0.23+/−0.004 µm/sec (p<0.001) after 1 h and to 0.17+/−0.003 µm/sec (p<0.001) after 2 h. However, in CDT-treated cells, microtubules and EB3 were associated for a longer period of time than in controls, indicating that capture, pause and/or catastrophe of microtubules were decreased (Figure 4D).

### Binary toxins influence distribution of CLASP2 and ACF7

**Figure 5:**
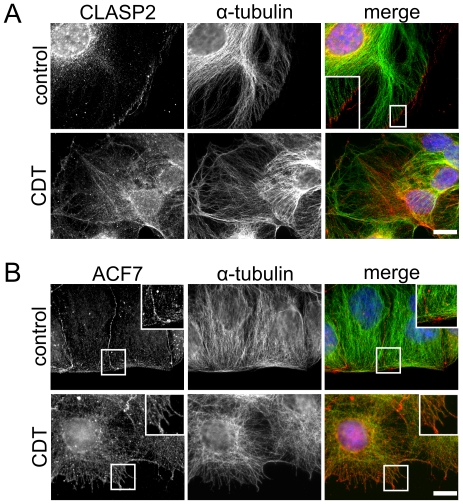
Influence of CDT on CLASP2 and ACF7 localization. (A) Indirect immunofluorescence of CLASP2 (red) and α-tubulin (green) in Caco-2 cells. The nucleus was stained by DAPI (blue). CLASP2 is translocated from the tip of microtubules that reached the cell cortex to filamentous structures in the cell interior. Cells were treated with 20 ng/ml CDTa and 40 ng/ml CDTb and fixed after 2 h. Scale bar represents 20 µm. (B) Indirect immunofluorescence of ACF7 (red) and α-tubulin (green) in Caco-2 cells. The nucleus was stained by DAPI (blue). ACF7 is translocated from the cell cortex to the microtubule lattice in the cell interior. Cells were treated with 20 ng/ml CDTa and 40 ng/ml CDTb and fixed after 2 h. White squares represent magnifications. Scale bar represents 20 µm.

We addressed the question whether proteins, which are suggested to be involved in capture of microtubules, were affected by actin-depolymerizing toxins. Besides EB1, CLASPs (CLIP- associated proteins) appear to participate in capture processes [33],[34]. As shown in Figure 5A, in controls CLASP2 is located at the cell cortex and binds to the tip of microtubules. After the treatment with actin-ADP-ribosylating toxin, CLASP2 translocated from the cell cortex and associated with filament structures, which were at least partially stained with anti-tubulin antibody (Figure 5A).

EB1 interacts with the large spectraplakin ACF7 (actin crosslinking family 7; also termed MACF1, microtubule and actin crosslinking factor 1) that functionally links microtubules to actin microfilaments [35]. After the treatment with CDT (or with C2 toxin) the distribution pattern of ACF7 changed. ACF7 was released from the cell cortex and associated with the microtubule lattice (Figure 5B). Thus, our data indicate that the actin-ADP-ribosylating toxin interferes with the capture of microtubules at the cell cortex by causing redistribution of proteins involved in capture processes.

**Figure 6:**
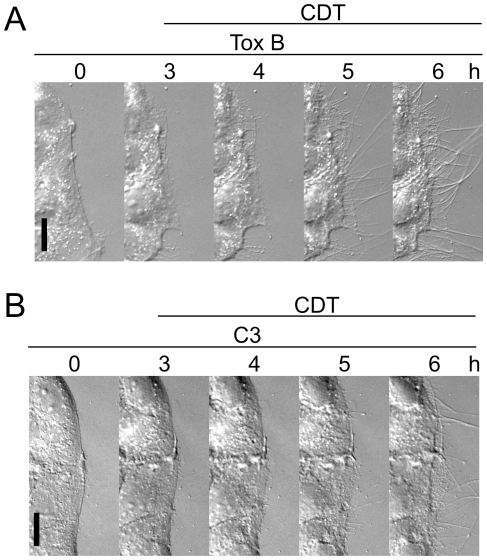
Influence of the Rho-inactivating toxin B and C3 toxin on CDT-induced protrusion formation. (A) Series of DIC time-lapse images of cells treated with 300 ng/ml toxin B at time point 0 h. After 3 h, 20 ng/ml CDTa and 40 ng/ml CDTb were added. Incubation was continued for 6 h. CDT induced the formation of microtubule-based protrusions after toxin B intoxication. Scale bar represents 20 µm. (B) Series of DIC time-lapse images of Caco-2 cells treated with 300 ng/ml C3 fusion toxin at time point 0 h. After 3 h, 20 ng/ml CDTa and 40 ng/ml CDTb were added. The incubation was continued for 6 h. CDT induced the formation of microtubule-based protrusions after C3 intoxication. Scale bar represents 20 µm.

Because interaction of ACF7 with F-actin and microtubules appears to be controlled by the small GTPase RhoA and its effector mDia [35], we studied the effects of the Rho-inactivating *C. difficile* toxin B on the formation of CDT-induced microtubule protrusions (Figure 6A). Treatment of Caco-2 cells with toxin B (300 ng/ml) for 2–4 h induced major morphological changes such as shrinking of the cell body. However, addition of CDT caused the same formation of protrusions as observed in the absence of toxin B. Also the pretreatment of cells with the Rho-inactivating toxin C3 (Figure 6B), which was applied as a C2IN-C3 fusion protein together with the binding component C2II, did not affect CDT-induced outgrowth of protrusions. These findings suggest that Rho GTPases do not play a major role in the effects caused by CDT.

### Formation of protrusions increases adherence of bacteria

**Figure 7:**
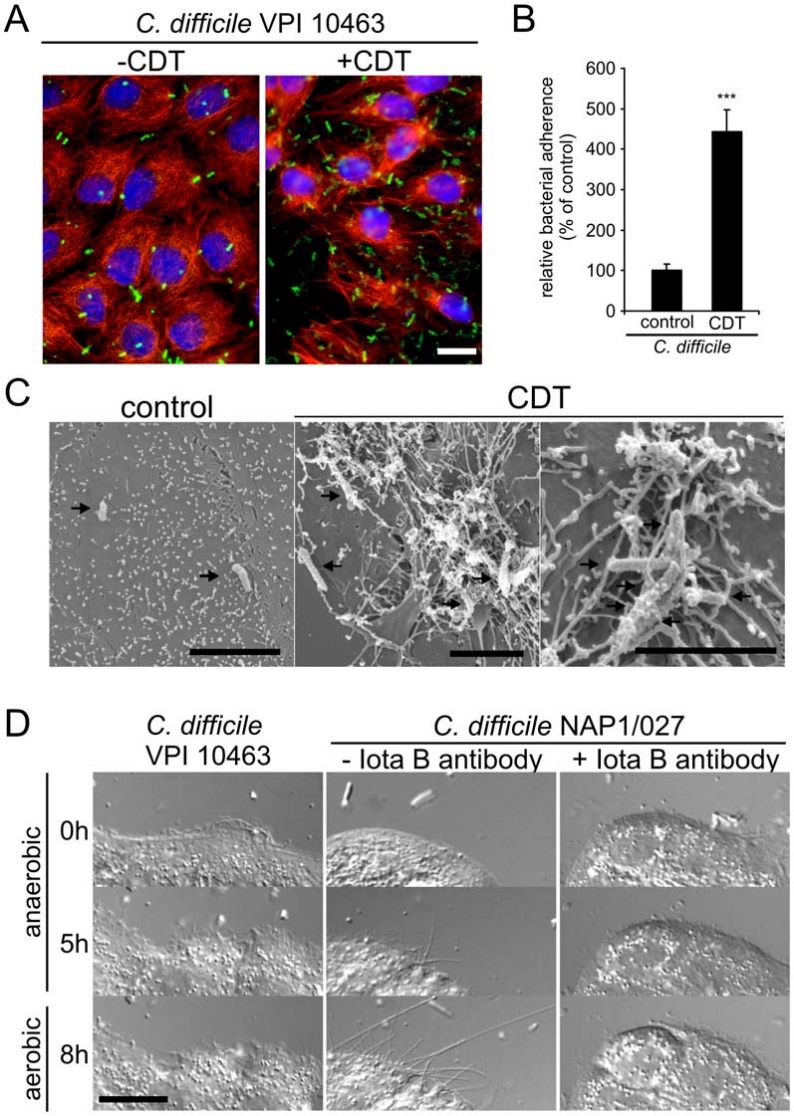
CDT treatment increases the adherence of *C. difficile*. (A) Indirect immunofluorescence of *C. difficile* surface proteins (green) and α-tubulin (red) on Caco-2 cells with adhered bacteria. The nucleus was stained by DAPI (blue). Cells were treated without and with 20 ng/ml CDTa and 40 ng/ml CDTb. After 1 h, 25 µl of an inoculum of *C. difficile* VPI 10463 (over night culture, OD 1.2, 2.5×10^8^ bacteria/ml) was added to a 24-well dish of confluent Caco-2 cells on a coverslip. After 90 min cells were washed and fixed. CDT intoxication increased the adherence of *C. difficile* VPI 10463. Scale bar represents 20 µm. (B) CDT also increased the adherence of *C. difficile* VPI 10463 under anaerobic conditions. 100 µl of a C. *difficile* over night culture (OD 1.2, 2.5×10^8^ bacteria/ml) was added to a 3 cm dish of confluent Caco-2 cells treated without or with 20 ng/ml CDTa and 40 ng/ml CDTb. The cells were incubated under anaerobic conditions for 4 h. After incubation the cells were washed, scraped off and plated. After 2 days CFUs were counted. Adherence after CDT treatment was quantified as percent of adherence on untreated cells. (C) Scanning electron microscopy of Caco-2 cells. Cells were treated as in Figure 7A. Scale bar represents 5 µm. After CDT treatment *Clostridia* were caught and wrapped in protrusions (arrows) (D) Time-lapse images of Caco-2 cells incubated together with the indicated strains of *C. difficile* for the indicated times. Cells were incubated for 5 h under anaerobic conditions (100% N_2_) and subsequently incubated under aerobic conditions (6.5% CO_2_ and 9% O_2_) for further 3 h. CDTb was neutralized by addition of anti-Iota B antibody (1∶50) at time point 0 h (right panel). CDT-producing *Clostridia* induced the formation of protrusions. A CDT-neutralizing antibody inhibited the formation of protrusions by CDT-producing *Clostridia*. Scale bar represents 20 µm.

To address the question whether the formation of the microtubule meshwork may affect adherence and/or colonization of bacteria, we incubated Caco-2 cells in the presence of CDT with *C. difficile* strain VPI 10463, which produces toxins A and B but not the actin-ADP-ribosylating toxin CDT. Cells were treated for 1 h with CDT, then *C. difficile* bacteria were added and the incubation was continued for 90 min. Thereafter, cell monolayers were washed and bacteria, which remained adherent to the cell surface, were determined by an antibody directed towards *C. difficile* surface proteins. As shown in Figure 7A, treatment with CDT largely increased the number of bacteria adherent to cells. About 6-fold higher numbers of bacteria were counted in the presence of CDT as compared to controls. Because cell adherence studies with *C. difficile* were not performed under anaerobic condition, which is important for development of the bacteria, we repeated the experiments under anaerobic conditions. For this purpose polarized Caco-2 cell mono-layers infected with bacteria were placed in an anaerobic incubation chamber for 4 h. This incubation under anaerobic conditions did not cause visible defects of the cell monolayer. Thereafter, cell monolayers were washed and removed from the culture dishes. The cell suspensions, containing bacteria, were replated on blood agar plates and incubated under anaerobic conditions. Colony forming units were determined after 2 days, resulting in an ∼4.5 fold increase with CDT as compared to controls (Figure 7B). Electron microscopic studies revealed that bacteria were surrounded and wrapped by protrusions formed in the presence of CDT (Figure 7C).

In the above mentioned experiments, purified CDT was added to cell culture and then adherence of *Clostridia* was studied. Therefore, we wanted to know whether production of CDT by *Clostridia* immediately affects adherence and colonization of the pathogen. To this end, Caco-2 cells were co-cultured with CDT-producing *C. difficile* strain Nap1/027 under anaerobic conditions for 5 h followed by aerobic incubation for further 3 h. Time-lapse microscopy revealed the formation of microtubule-based protrusions after 5 h, which largely increased within the next 3 h (Figure 7D). The production of CDT under these conditions was verified by testing the ADP-ribosylating activity in the culture supernatant (data not shown).

By contrast, a *C. difficile* strain (VPI 10463) that does not produce CDT was not able to induce protrusions. Moreover, addition of anti-iota B antibody, which is known to interact with the binding component of CDT [36], prevented the formation of protrusions caused by *C. difficile* Nap1/027, indicating that the formation of protrusions depended on the actin ADP-ribosylating toxin CDT (Figure 7D). To verify the importance of CDT in the establishment of disease, we employed an *in vivo* model of antibiotic-associated diarrhea.

In general, mice are resistant against *C. difficile* infection and do not develop any signs of disease (i.e. gut inflammation, colitis). For a long time, attempts to establish a mouse model for *C. difficile*-mediated antibiotic-associated diarrhea have yielded ill-reproducible results. Most likely, mouse-colony specific differences in gut flora composition have been responsible for the poor reproducibility. Only very recently, antibiotic treatment regimes have been developed which allow re-capitulating the hallmarks of antibiotic-facilitated *C. difficile* colitis in conventional mice [37],[38]. To minimize interference by components of the normal gut flora, we used gnotobiotic mice colonized with the Altered Schaedler Flora (ASF) [39] for *C. difficile* infection. Mice were treated with clindamycin (0.2 mg) 24 h previous to infection with the human pathogenic *C. difficile* strain Nap1/027 (10^7^ CFU). To investigate the role of CDT in enteric infection, we neutralized CDT in Nap1/027-infected mice by oral treatment with CDT-neutralizing rabbit anti-iota antiserum. The other group of mice was treated with control serum. Notably, in contrast to mice treated with CDT-neutralizing anti-iota toxin antibody, mice treated with the control serum developed signs of diarrhea (liquid stool) at 24 h after infection. Mice were sacrificed 30 h after infection. Upon macroscopic inspection, a great part of the control mice exhibited signs of colitis and approximately half of the mice showed overt inflammation of the cecum (data not shown). As the key experimental readout, we analyzed intestinal colonization by Nap1/027. *C. difficile* colonized anti-iota toxin treated mice at significantly lower levels as compared to control mice (Figure S7).

Next, we analyzed *in vivo* epithelial adherence of *C. difficile* by immunofluorescent staining with a specific *C. difficil*e antiserum. In CDT-neutralizing anti-iota toxin serum treated mice immunohistochemical stainings of sections of colon and cecum, revealed a lower number of bacteria directly adjacent to the mucosal surface (Figure S8A–C).

Altogether, our data suggest that CDT increases epithelial adherence *in vitro* and *in vivo*, thereby supporting bacterial colonization.

## Discussion

Here we report on a novel morphological feature induced by binary actin-ADP-ribosylating toxins, which modify actin at Arg-177, and thereby inhibit F-actin formation and cause microfilament depolymerization. We show that *C. difficile* CDT, *C. botulinum* C2 toxin and *C. perfringens* iota toxin induce cellular protrusions in epithelial cells, which are thin, highly dynamic structures with lengths from 5 to >150 µm. Immunofluorescence microscopy revealed that the protrusions harbored dynamic microtubules. Formation of protrusions occurred concomitantly with ADP-ribosylation of actin and depolymerization of microfilaments. However, complete depolymerization of the actin cytoskeleton was not necessary for the formation of protrusions. The microtubule structures were formed when only a small part of actin was ADP-ribosylated and cell morphology was not grossly altered. Moreover, major formation of microtubule protrusions was observed at medium concentration of the toxins. It appeared that at rather high concentrations of CDT, formation of microtubule-based protrusions occurred rapidly but their elongation ceased soon.

Treatment of cells with the actin-depolymerizing toxins not only induced formation of microtubule protrusions but also changed cellular microtubule structures. Increased bundling of microtubules was observed. Previously it was reported that the actin-ADP-ribosylating C2 toxin affects microtubule patterns in human leukemia cells by causing formation of microtubule bundles [40]. In addition actin filament-depolymerizing agents like cytochalasin and latrunculin, which directly interact with actin to interfere with actin polymerization, can induce microtubule-based cell surface extensions [41],[42]. However, these compounds were much less effective than the binary actin-ADP-ribosylating toxins. On the other hand, we observed that the F-actin-stabilizing compound jasplakinolide caused delay in the action of the binary toxins. Filamentous actin is a poor substrate for ADP-ribosylation by binary toxins, which may explain the delay of the action of CDT in the presence of jasplakinolide. Moreover, treatment with jasplakinolide may delay the release of microtubule-capturing proteins from the cell cortex (see below).

Microtubule dynamics are regulated by a variety of microtubule associated proteins. A major player in the regulation of microtubule dynamics is EB1, which promotes microtubule polymerization by increasing the number of rescue events and decreasing the rate of depolymerization or the time in pause [29],[43],[44]. Since almost all other +TIPs additionally interact with EBs [45], we decided to study the influence of actin–ADP-ribosylating toxins on the distribution of EB1. We showed that toxin treatment increased the length of EB1 comets at the growing tips of microtubules. Additionally, by the use of live imaging we showed that EB3-comets were present for longer time periods after toxin treatment, indicative of longer growth phases of microtubules. Surprisingly, these EB1-comets revealed a decrease in microtubule polymerization speed.

Capture of growing microtubules at the cell cortex is an important process in the control of microtubule dynamics. Several proteins have been suggested to be involved in microtubule capture besides EB1 at least CLASP1/2, and ACF7 appear to play a role [33]–[35],[46]. We observed that CDT treatment of Caco-2 cells caused redistribution of CLASP2 and ACF7 from the cell cortex and microtubule ends to the cell interior. These findings are in line with the notion that microfilaments are essential for the localization of these proteins at the cell cortex and depolymerization of actin causes redistribution of these proteins. The functions of ACF7 have been studied in ACF7−/− mouse embryonic fibroblasts, showing that deletion of ACF7 causes defects in microtubule capture with bending of microtubules at the cell cortex. We also observed bending of microtubules after CDT treatment; however, the main effect of the toxin was the formation of protrusions. This was not described by deletion of ACF7 [35]. Importantly, the deletion studies were performed in the presence of functional microfilaments, whereas in our experiments the polymerization of actin is inhibited by the toxins.

So far the functional link between microfilaments and the microtubule system is still enigmatic. Master regulators of the actin cytoskeleton belong to the family of Rho GTPases, including Rho, Rac and Cdc42 proteins [47]–[49]. These switch proteins control actin polymerization by regulating the activity of the formin proteins mDia [50], or Wasp and Scar/Wave proteins, respectively, which control actin polymerization by Arp2/3 complexes [51]. Recently many studies showed that Rho GTPases play also a major role in controlling microtubules [52]. For example, RhoA regulates microtubules via mDia [53],[54]. This effect appears to be independent of the action of mDia on actin [55]. Rac reportedly controls microtubules by stathmin phosphorylation via DOCK7 [56] and also Cdc42 is implicated in regulation of the microtubule system [57]. Another example is IQGAP, which interacts with Rac and Cdc42, and was shown to associate with microtubules via CLIP-170 [58]. However, our studies with *C. difficile* toxin B exclude the involvement of Rho, Rac or Cdc42 in toxin-induced formation of microtubule protrusions. Treatment of cells with toxin B, which inhibits the GTPases [3], did not prevent subsequent induction of formation of protrusions by CDT. Notably in these studies, rather high concentrations of toxin B were used and the incubation was continued until major cellular shrinking was observed, indicating full action of toxin B. Recently, it has been reported that inhibition of myosin II, which was shown to be involved in epithelial cyst formation, causes depolymerization of F-actin bundles and formation of cellular processes, which resemble in size and microtubule content our toxin-induced protrusions [59] but do contain a large part of F-actin, indicating that these protrusions are different. Ivanov and coworkers reported that phospholipase C might be involved in the process formation [59]. Until now we could not prove an involvement of phospholipase C in the formation of the reported microtubule-based protrusions.

CDT-induced formation of protrusions increased the adherence of *C. difficile* at the cell surface of epithelial cells. Electron microscopical studies revealed that the protrusions formed a dense meshwork in which the *Clostridia* were caught, suggesting that CDT has major effects on the colonization of *Clostridia*. In fact it has been suggested that the toxin favors colonization of *C. difficile* bacteria [60]. Besides toxin A and B, which both glucosylate Rho GTPases, CDT is the third toxin produced by *C. difficile*. It is generally accepted that toxins A and B are the cause of pseudomembranous colitis induced by the pathogen [2]–[4],[61]. However, recently identified hypervirulent strains of *C. difficile* (e.g., ribotype 027/Nap1) also produce CDT and the number of isolates, producing CDT, is increasing [7]–[11]. The role of the additional toxin CDT as a virulence factor was not clear so far. It has been reported that CDT-producing strains, which do not produce the glucosylating toxins A and B (A^−^ B^−^ CDT^+^-strains), did not cause disease but colonize in hamster after challenge with clindamycin. On the other hand, A^+^B^+^CDT^−^-strains can colonize and cause disease under the same conditions [60].

Our findings show that the toxin has unexpected effects on the morphology of intestinal cells, which directly affect the environment of the *Clostridia*. We show that CDT induces a ∼5 fold increase in adherence of *C. difficile* bacteria under anaerobic conditions. Moreover, *in vivo* studies in mice were in line with these results. When mice were infected with a CDT-producing strain, we observed a 4 fold increase in epithelial adherence of bacteria in the large intestine as compared to mice treated with CDT-neutralizing antiserum.

These data were corroborated by the finding that *C. difficile* colonization of the cecal content was significantly decreased when CDT was functionally neutralized in the gut. Considering the massive effects of CDT on the microtubule system and the formation of a meshwork of microtubule-based protrusions tightly covering bacteria at the surface of intestinal epithelial cells, we propose that at least one major effect of CDT is increased epithelial adherence and thereby optimization of colonization of the pathogen. It was shown that *C. difficile* binds to extracellular matrix proteins like fibronectin or collagen [62]. Binding to extracellular matrix proteins appears to occur at least in part via surface layer proteins (SLPs) of *C. difficile* [63]. Also flagellar proteins are implicated in adherence of *C. difficile* [64]. It remains to be studied, which bacterial surface structures are involved in interaction with these cellular extensions. It has been suggested that interaction of *Clostridia* with extracellular matrix proteins is not possible before major alteration of epithelial cells are induced by cytotoxins. Thus, in the early phase of infection CDT-induced formation of protrusions might be of special importance for adherence of *Clostridia*.

Taken together, our findings suggest an important role of microtubule-based protrusions in enhancing cell surface adherence and colonization by *C. difficile*. Hence our findings of the toxin effects, which are shared by all actin ADP-ribosylating toxins studied, have a major impact in understanding the role of these toxins produced by several *Clostridia*. Different pathogenic *Clostridia* might exploit this mechanism by the production of ADP-ribosylating toxins. *C. botulinum* type C and D produces in addition to the highly potent neurotoxins, the actin modifying C2 toxin. For this reason, C2 might be of relevance for botulism in waterfowl [65]. Therefore, our studies not only demonstrate an important functional connection between actin microfilaments and regulation of microtubules but also offer a new perspective in understanding the role of the binary actin-modifying toxins in infectious biology and host-pathogen interactions.

## Materials and Methods

### Actin-affecting compounds

Cytochalasin D was obtained form Sigma (St. Louis, MO), latrunculin B from Calbiochem (Merck, Darmstadt, Germany) and jasplakinolide from Axxora (Lörrach, Germany).

### Expression and purification of protein toxins

The components of C2 toxin (C2I and C2II) [66] and of iota toxin (Ia and Ib) [67] were purified as described.

The components of CDT (CDTa and CDTb) were purified as recombinant glutathione S-transferase proteins. GST-Proteins were expressed in *E. coli* TG1 pACYC IRL10 cells (CDTa) and in *E. coli* BL21 DE3 Rosetta cells (CDTb). The proteins were purified by affinity chromatography with glutathione-Sepharose 4B, according to the manufacturer's instructions. Glutathione S-transferase was cleaved off by thrombin (3.25 NIH units/mL of bead suspension). The inactivation of thrombin was performed by the addition of benzamidine beads. CDTb was activated by 0.2 µg of trypsin/µg of protein for 30 min at 37°C.

All binding components used (CDTb, C2II and Ib) mentioned in the text were used as protease-activated proteins according to [66],[67].

Purification of the Rho-inactivating C3 toxin, which was used as a cell permeable fusion toxin, has been described [68].

*C. difficile* (strain VPI 10463) toxin B was purified as described [69].

### Construction of CDTb expression plasmid

pGEX-CDTa construction was described previously [20]. For construction of pGEX2T-CDTb, *cdtb* (2631 bp) was amplified by PCR, using genomic DNA from *C. difficile* strain 196 (gift from M. Popoff, Institute Pasteur, Paris) as a template. Used primers: 5′CDTb-*Bgl*II GGG GGG AGA TCT ACC ATG AAA ATA CAA ATG AGG AAT AAA AAG G and 3′CDTb-*Eco*RI GGG GGG GAA TTC CTA ATC AAC ACT AAG AAC TAA TAA CTC. The PCR-product was cloned into the pGEX-2T-vector.

### Antibodies and fluorescent dyes

Mouse anti-EB1 and anti-EB3 antibodies were from BD Biosciences (Franklin Lakes, NY), mouse monoclonal anti-α-tubulin, anti-acetylated tubulin antibody were from Sigma (St. Louis, MO), mouse anti-detyrosinated tubulin antibody was from Millipore (Billerica, MA). Anti-tyrosinated tubulin antibody YL1/2 was used as described before [70]. Mouse anti-MACF1/ACF7 antibody from Abnova (Heidelberg, Germany). Rabbit anti-MACF1/ACF7 antibody and anti-occludin antibody was from Santa Cruz (Santa Cruz, CA). Anti-ZO-2 antibody was from Cell Singnalling Technology (Beverly, MA). Anti-CLIP-170 antibody [31], anti-CLIP-115 antibody [32] and anti-CLASP2 antibody [34] were a gift from Dr. Niels Galjart. As secondary antibodies Alexa568- and Alexa488-conjugated anti-rabbit, anti-mouse and anti-rat antibodies (Invitrogen, Karlsruhe, Germany) were used. Nuclei were stained by DAPI-Prolong Gold and filamentous actin was stained using phalloidin-TRITC (Invitrogen, Karlsruhe, Germany). Antisera against *C. difficile* 630 were from Dr. Neil Fairweather (Imperial College, London).

### Cell culture, transient transfection

Caco-2 cells were cultured in Dulbecco's modified Eagle's medium supplemented with 10% fetal calf serum (FCS), 1% non essential amino acids and 1% Na-pyruvate, all from Biochrom (Berlin, Germany). HT29 cells were cultured in Mc Coy's 5A Medium (Pan Biotech Aidenbach, Germany) supplemented with 10% FCS. For immunostaining, cells were plated on HCl-washed coverslips. For life cell imaging, cells were plated on glass bottom dishes (Mattek, Ashland, MA). Cells were transfected using Lipofectamine 2000 (Invitrogen, Karlsruhe, Germany) according to the manufacturer's protocol.

### Bacterial culture

*C. difficile* strain VPI 10463 was cultured in Brain Heart Infusion medium (BD, Franklin Lakes, NY) at 37°C under anaerobic conditions (Anaerocult A, Merck, Darmstadt, Germany). For quantification of bacterial adherence, 100 µl of a C. *difficile* over night culture (OD 1.2; 2.5×10^8^ bacteria/ml) were added to a 3 cm dish of polarized Caco-2 monolayer, containing 2 ml cell culture medium with 15 mM HEPES-Buffer (Biochrom, Berlin, Germany). The cells were incubated under anaerobic conditions for 4 h. After incubation the cells were washed, removed and plated on blood agar plates. After 2 days colony forming units (CFUs) were counted. Adherence of *Clostridia* after CDT treatment was quantified as percent of adherence on control cell monolayer.

### ADP-ribosylation assay

ADP-ribosylation was performed at different time points after toxin treatment. 30 µg of whole-cell lysate were used. The subsequent ADP-ribosylation reaction was performed for 30 min at 30°C with 300 ng of C2I in a buffer, containing 20 mM Tris-HCl (pH 7.5), 1 mM EDTA, 1 mM dithiothreitol, 5 mM MgCl_2_, and Complete protease inhibitor (Roche, Basel, Switzerland)(according to the manufacturer's manual). The reaction was stopped by boiling the sample with SDS-sample buffer. The samples were run on SDS-PAGE, and [^32^P]ADP-ribosylated proteins were detected by autoradiography with a PhosphorImager (Amersham Biosciences, Freiburg, Germany).

### Immunostaining, measurement of +TIP length

Cells were washed with warmed PBS, fixed in ice-cold methanol, containing 1 mM EGTA (left out for phalloidin-TRITC staining), postfixed with 4% formaldehyde, and permeabilized with 0.15% Triton X-100 in PBS. Cells were blocked by 1% BSA and 0.05% Tween 20 in PBS. Incubation with first antibody was performed over night at 4°C in 1∶200, 1∶300 or 1∶1000 dilutions in blocking buffer dependent on the antibodies. Then, samples were washed with 0.05% Tween 20 in PBS and incubated with 1∶200 dilutions of the secondary antibodies for 1.5 h at room temperature. Thereafter, cells were washed again and embedded with Prolong Gold. Fixed samples were analyzed by fluorescence microscopy, using an upright Zeiss Observer microscope system (Carl Zeiss GmbH, Jena, Germany) equipped with an apotom. For the measurement of +TIP length, 100 +TIPs in each group were analyzed with Metamorph software (Universal Imaging, Downingtown, PA).

### Live cell imaging and quantification of formation of protrusions and +TIP dynamics

For live cell imaging cells were observed in a chamber that provided a humidified atmosphere (6.5% CO2 and 9% O2) at 37°C on a Zeiss Axiovert 200 M inverted microscope (Carl Zeiss GmbH, Jena, Germany). Specific fluorescence illumination was generated by a monochromator (Cairn Optoscan; Faversham Kent, UK). Images of 16-bit depth were collected with a digital camera (Coolsnap HQ; Roper Scientific, Tucson, AZ) driven by Metamorph imaging software (Universal Imaging, Downingtown, PA). For the observation of formation of microtubule protrusions time-lapse series, lasting 2–8 h, were acquired with 90 sec intervals. For quantification of formation of microtubule protrusions, the lengths of all processes were summated every 15 min and normalized by the respective section of cell perimeter (an example of quantification of protrusion formation is given in Figure S2).

For the observation of +TIP dynamics cells were transiently transfected with an EB3-GFP expression vector [71] (kind gift of Dr. N. Galjart) and imaged 24–48 h after transfection. Only cells with low expression levels were employed. Time-lapse series with 2–4 min duration were acquired with a 2 sec interval. Control cells were imaged and after treatment for 1 and 2 h, the same cells were imaged again. All quantifications were performed by the Metamorph software.

For the quantification of microtubule polymerization speed, 100 steadily moving +TIPs for each group were tracked for 10 sec. The rate of polymerization was calculated by the covered distance during this time. For the quantification of the time in growth, 30 microtubules with EB3-GFP association at tips of microtubules were recorded from their appearance until fading.

In live cell imaging experiments photo-bleaching and photo-stress were reduced by minimal aperture of the monochromator exit slit and the microscope was equipped with high speed shutters for fluorescence and transmitted light illumination.

### Preparation of primary colon epithelial cells

The colon of a Wistar rat was excised and transferred to ice-chilled PBS, containing 50 µg/ml penicillin/streptomycin (Biochrom, Berlin, Germany). The colon was washed three times. Ions were chelated by 1 mM EDTA and 1 mM EGTA for 1 h at 21°C. To release the epithelial cells from the lamina propria, the tube was vigorously shaken. The piece of tissue was removed and the liberated epithelial cells were pelleted at 50 g for 3 min. The cells were washed in growth DMEM medium (supplemented with 15% FCS, 1% Na-pyruvate, 1% non essential amino acids and 50 µg/ml penicillin/streptomycin). Subsequently, cells were plated on collagen-coated dishes or coverslips. Experiments were performed after 1–2 days *in vitro*.

### Scanning electron microscopy

For scanning electron microscopy of cells the cultivation medium was replaced by ice-cold cacodylate buffer (0.1 M cacodylate, 0.01 M CaCl_2_, 0.01 M MgCl_2_, 0.09 M saccharose, pH 6.9) and prefixed by adding paraformaldehyde to a final concentration of 5%, after 10 min glutaraldehyde was added to a final concentration of 2%. Cells were dehydrated by incubation in a graded series of acetone in 10% steps to 100%, critical-point dried with liquid CO_2_ (CPD030, Bal-Tec, Liechtenstein) and covered with a thin gold film for conductivity by sputter coating (SCD040, Balzers, Liechtenstein or Cressington 108 auto, Watford, UK). Samples were examined in a field emission scanning electron microscope DSM982 Gemini at an acceleration voltage of 5 kV or in a LEO 435VP (Carl Zeiss SMT, Oberkochen, Germany) at an acceleration voltage of 10 kV.

### Animals

Gnotobiotic mice were created by colonizing germfree Swiss Webster mice with a donor mouse, associated with the *Altered Schaedler Flora* (ASF), according to the protocol published on the Taconic webpage (Taconic, Hudson, NY, www.taconic.com/library). Gnotobiotic mice were maintained under barrier conditions in individually ventilated cages with autoclaved chow and autoclaved, acidified water.

### Infection of mice

24 h previous to infection with *C. difficile* Nap1/027, mice were pretreated with 0.2 mg clindamycin (Clinda-saar, MIP Pharma) by gavage. Mice were infected by gavage with 10^7^ CFU *C. difficile* Nap1/027 in 0.2 ml PBS. Mice were treated with anti-iota or control serum (100 µl in case of the first animal experiment and 200 µl in the second experiment) by oral gavage directly after infection (or at 7 h postinfection with similar results) and at 24 h postinfection. To inactivate complement, serum was incubated for 30 min at 56°C. 30 h post infection, mice were sacrificed by cervical dislocation.

The experiments were performed on 2 independent occasions with a total of 10 Nap1/027 infected mice treated with anti-iota CDT-neutralizing serum, 11 Nap1/027 infected mice treated with control serum and 4 control mice. 2 of the control mice were also treated with control serum to exclude a serum induced inflammation.

For histology, intestinal tissue was cryo-embedded in Tissue Tek OCT (Sakura) and flash frozen. For immunofluorescent staining, the tissue was fixed in 4% PFA over night followed by over night equilibration in 20% sucrose and cryo-embedding. Cryosections (7 µm) of PFA-fixed and cryo-embedded tissue from cecum and colon were mounted on glass slides and air dried for 2 h at room temperature prior to immunostaining. Sections were fixed again in 4% paraformaldehyde for 5 min, washed and blocked with 1% BSA in PBS. The primary antibody was detected with an Alexa 488 labeled anti-rabbit antibody. The sections were incubated over night with a rabbit anti-*C. difficile* serum. Actin was stained by TRITC-phalloidin and the nuclei by DAPI. For bacterial quantification, bacteria adjacent to the epithelium, stained with anti-*C.difficile* serum, within 3 µm of the epithelium were counted. If bacterial aggregations were attached to the epithelium all bacterial cells in the aggregation were counted. This was done for ≥15 optical fields per group. The number of *C. difficile* per µm mucosa surface was determined.

For bacteriology, cecal content was removed aseptically and homogenized in 4°C PBS. The bacterial loads in cecal content were determined by plating on *C. difficile* selective agar plates (Oxoid, Basingstoke, UK) at different dilutions. The minimal detectable value was 10 CFU/g.

### Statistics

Student's t test was used when two groups of parametric data with normal distribution had to be compared. The Mann-Whitney U test was applied for processed data without normal distribution. Statistic evaluation was performed with the Sigma Stat software (Jandel Scientific, Corte Madera, CA) or with Prism 4 (GraphPad, La Jolla, CA). P values <0.05 were considered statistically significant.

### Ethics statement

Animal experiments were approved (license 201/2004 and 201/2007 Kantonales Veterinäramt Zürich) and performed as ethically and legally required.

## Supporting Information

Figure S1 Specificity of toxin-induced formation of protrusions. (A) Both CDT components are necessary for protrusion formation. Series of DIC time-lapse images of Caco-2 cells treated with 20 ng/ml CDTa or 40 ng/ml CDTb. Subconfluent Caco-2 cells were treated with each toxin component for 0, 2 and 5 h. No formation of protrusions was observed when only one toxin component was added. Bar, 20 µm. (B) A catalytic inactive mutant of CDTa E430Q did not induce protrusion formation. DIC time-lapse microscopy of Caco-2 cells. Subconfluent Caco-2 cells were treated with 20 ng/ml CDTa E430Q and 40 ng/ml CDTb. No formation of protrusions was observed. (C) The ADP-ribosylating toxins iota toxin and C2 toxin induce formation of protrusions. DIC time-lapse microscopy of Caco-2 cells. Subconfluent Caco-2 cells were treated with 100 ng/ml iota toxin Ia (enzyme component) and 200 ng/ml iota toxin Ib (binding component) (iota, left panel) or with 250 ng/ml C2I and 500 ng/ml C2II (C2, right panel) for 6 h. Bar, 20 µm. (D) Primary colonocytes from the rat gut form protrusions after toxin treatment. DIC time-lapse microscopy of primary rat colonocytes after 1.5 days in vitro. Rat colonocytes were prepared as described in the Methods section and cultured for 1.5 days on a collagen-coated glass surface. The primary cells were treated with 20 ng/ml CDTa and 40 ng/ml CDTb. Pictures were taken after 0, 45, 75 and 90 min, respectively. In each panel the incubation time (h∶min) is indicated. Bar, 10 µm.
(0.71 MB PDF)

Figure S2 Quantification method of toxin-induced formation of protrusions. The lengths of protrusions (indicated 1–5) were determined by using the Metamorph software and the sum normalized by the respective section of cell perimeter (indicated as dashed line). Here Caco-2 cells were used and the toxin concentration was 20 ng/ml CDTa and 40 ng/ml CDTb. Incubation time was 1 h.
(0.05 MB PDF)

Figure S3 Visualization microtubule-based protrusions on polarized Caco-2 cells. (A) Caco-2 cells were grown on filters for 2 weeks to ensure polarization. Cells were treated with 20 ng/ml CDTa and 40 ng/ml CDTb. Untreated control cells show microvilli at the cell surface. On CDT-treated cells, microvilli disappeared and showed pronounced formation of protrusions. Scale bar represents 10 µm. (B) Confluent Caco-2 cells were grown for 1.5 weeks. The polarized cells were treated with 20 ng/ml CDTa and 40 ng/ml CDTb and fixed after 2 h or remained untreated as control cells. Confocal images of indirect immunofluorescence of α-tubulin and occludin were acquired as a Z-stack. Yellow arrows mark microtubule bundles at the cell surface. The relative position of the picture in the Z-stack is indicated on the left. Scale bar represents 10 µm. (C) Confluent Caco-2 cells grown for 1.5 weeks (as in S3B). The polarized cells were treated with 20 ng/ml CDTa and 40 ng/ml CDTb and fixed after 2 h or remained untreated as control cells. Confocal images of indirect immunofluorescence of α-tubulin and ZO-2 were acquired as a Z-stack. Yellow dashed lines represent cell borders delineated according to the ZO-2 staining. The relative position of the picture in the Z-stack is indicated on the left. The real position is shown in the cut view below. The cut view is reconstructed along the red line in the upper picture of the Z-stack. CDT treated cells are higher. As a result the area of the nucleus is not represented in the shown Z-stack of CDT-treatment. Scale bar represents 10 µm. (D) Magnification of the white square in Figure S3C (right panel), showing the microtubule-based protrusion meshwork at the cell surface.
(3.07 MB PDF)

Figure S4 Staining of posttranslationally modified tubulin. Subconfluent Caco-2 cells were treated with 20 ng/ml CDTa and 40 ng/ml CDTb and fixed after 2 h or remained untreated as control cells. (A) Indirect immunofluorescence of α-tubulin (green) and detyrosinated tubulin (Glu-tubulin) (red). The amount of Glu-tubulin is not increased. Protrusions are not formed by Glu-tubulin. (B) Indirect immunofluorescence of α-tubulin (green) and acetylated tubulin (red). The amount of acetylated tubulin is not increased. Protrusions are not formed by actetylated tubulin. (C) Indirect immunofluorescence of α-tubulin (green) and tyrosinated tubulin (Tyr-tubulin) (red). The protrusions are formed by dynamic tyrosinated tubulin. Scale bar represents 20 μm (A,B,C).
(2.19 MB PDF)

Figure S5 Influence of actin stabilizing and destabilizing drugs on the formation of microtubule-based protrusions. (A) Series of time-lapse images of cells treated with 20 ng/ml CDTa and 40 ng/ml CDTb, 5 µM latrunculin B and 1 µM cytochalasin D, respectively. Scale bar represents 20 µm. Actin destabilizing drugs can also induce protrusions, but less effective compared to CDT. (B) Series of time-lapse images of cells, which were treated with 500 nM jasplakinolide (jas) for 30 min. Then 20 ng/ml CDTa and 40 ng/ml CDTb were added and the formation of protrusions was monitored. Scale bar represents 20 µm. Actin stabilization can delay and decrease the formation of protrusions. (C) DIC time-lapse microscopy of Caco-2 cells. Subconfluent Caco-2 cells were treated with 300 ng/ml toxin B (Tox B) or 300 ng/ml C3 fusion toxin (C3) and 500 ng/ml C2II (to deliver the C3-fusion toxin into the cells) for the indicated times (h). The toxins were added to the cell culture medium. Incubation with toxins was continued until major morphological changes occurred. No formation of protrusions was observed under these conditions. Scale bar represents 20 µm.
(0.87 MB PDF)

Figure S6 Influence of CDT on EB3 and CLIP-115 proteins. Subconfluent Caco-2 cells were treated with 20 ng/ml CDTa and 40 ng/ml CDTb and fixed after 1 h. Indirect immunofluorescence pictures of EB3 (red) by using anti-EB3 antibody and CLIP-115 (green) by using anti-CLIP-115 antibody in Caco-2 cells are shown. The nucleus was stained by DAPI (blue). Scale bar represents 20 µm. The increase of EB3 and CLIP-115 comet length after toxin treatment is seen.
(0.84 MB PDF)

Figure S7 CDT increases bacterial loads of *C. difficile* in cecal content *in vivo*. Mice were infected by gavage with Nap1/027 (10^7^ CFU) and subsequently treated with control serum or CDT-neutralizing anti-iota toxin serum. Cecal content was aseptically removed and homogenized. Bacterial loads in the cecal content from uninfected untreated mice, Nap1/027 (10^7^ CFU) infected CDT-neutralizing anti-iota toxin serum treated mice and Nap1/027 infected control antiserum treated mice were determined by plating on *C. difficile* selective agar plates at different dilutions. The minimal detectable value was 10 CFU/g. The experiments were performed on 2 independent occasions with a total of 10 Nap1/027 infected mice treated with anti-iota CDT-neutralizing serum, 11 Nap1/027 infected mice treated with control serum and 4 control mice. Boxes indicate 25th and 75th percentiles, black bars indicate medians, and whiskers indicate data ranges. Y-axis is scaled logarithmically (log_10_). * indicates p≤0.05.
(0.03 MB PDF)

Figure S8 CDT increases adherence of *C. difficile* in the cecum and colon *in vivo*. (A) Mice were infected with Nap1/027 (10^7^ CFU) and subsequently treated with control serum or CDT-neutralizing anti-iota toxin serum. Cryosections (7 µm) of PFA-fixed and cryo-embedded tissue from cecum and colon were immunostained for *C. difficile*. Actin was stained by TRITC-phalloidin and the nuclei by DAPI. M marks the mucosa and L marks the lumen of the intestine. White boxes are magnified. White arrows mark bacteria or bacterial aggregations adjacent to the epithelium. Calibration bar represents 20 µm. (B) Quantification of *C. difficile* directly adjacent to the epithelium in the cecum. For bacterial quantification, bacteria adjacent to the epithelium, stained with anti-*C. difficile* serum, within 3 µm of the epithelium were counted. If bacterial aggregations were attached to the epithelium all bacterial cells in the aggregation were counted. This was done for ≥15 optical fields per group. The number of *C. difficile* per µm mucosal surface was determined. Nap1/027 infected mice treated with control serum were set 100%. (C) Quantification of *C. difficile* directly adjacent to the epithelium in the colon. The number of *C. difficile* per µm mucosa was determined as in B.
(0.85 MB PDF)

Video S1 DIC time-lapse microscopy movie of Caco-2 cells. Subconfluent Caco-2 cells were treated with 20 ng/ml CDTa and 40 ng/ml CDTb. The toxin was added to the cell culture medium. Thereafter, a picture was taken every 90 sec. After ∼50 min the formation of toxin-induced protrusions started. The maximum of protrusion formation was observed after ∼4 h. The incubation time (h∶min) is indicated in the movie. Scale bar represents 20 µm.
(6.92 MB MOV)

Video S2 Fluorescence time-lapse microscopy of Caco-2 cells transfected with EB3-GFP. Cells were treated with 20 ng/ml CDTa and 40 ng/ml CDTb for 2 h. The arrowhead marks the place where several EB3-GFP comets approach the cell cortex, cross the cell border and form a microtubule-based protrusion. Pictures were acquired every 2 sec. The elapsed time (min∶sec) is indicated in the movie. Contrast was inverted. Scale bar represents 5 µm.
(0.35 MB MOV)

## References

[1] Barbieri JT, Riese MJ, Aktories K (2002). Bacterial toxins that modify the actin cytoskeleton.. Annu Rev Cell Dev Biol.

[2] Voth DE, Ballard JD (2005). *Clostridium difficile* toxins: mechanism of action and role in disease.. Clin Microbiol Rev.

[3] Just I, Gerhard R (2004). Large clostridial cytotoxins.. Rev Physiol Biochem Pharmacol.

[4] Kelly CP, LaMont JT (2008). *Clostridium difficile*–more difficult than ever.. N Engl J Med.

[5] Bartlett JG (2002). Clinical Practice: Antibiotic-associated Diarrhea.. N Engl J Med.

[6] Just I, Selzer J, Wilm M, Von Eichel-Streiber C, Mann M (1995). Glucosylation of Rho proteins by *Clostridium difficile* toxin B.. Nature.

[7] Goncalves C, Decre D, Barbut F, Burghoffer B, Petit JC (2004). Prevalence and characterization of a binary toxin (actin-specific ADP-ribosyltransferase) from *Clostridium difficile*.. J Clin Microbiol.

[8] Geric B, Rupnik M, Gerding DN, Grabnar M, Johnson S (2004). Distribution of *Clostridium difficile* variant toxinotypes and strains with binary toxin genes among clinical isolates in an American hospital.. J Med Microbiol.

[9] Stubbs S, Rupnik M, Gibert M, Brazier J, Duerden B (2000). Production of actin-specific ADP-ribosyltransferase (binary toxin) by strains of *Clostridium difficile*.. FEMS Microbiol Lett.

[10] Martin H, Willey B, Low DE, Staempfli HR, McGeer A (2008). Characterization of *Clostridium difficile* Strains Isolated from Patients in Ontario, Canada, from 2004 to 2006.. J Clin Microbiol.

[11] McDonald LC, Killgore GE, Thompson A, Owens RC, Kazakova SV (2005). An epidemic, toxin gene-variant strain of *Clostridium difficile*.. N Engl J Med.

[12] Perelle S, Gibert M, bourlioux P, Corthier G, Popoff MR (1997). Production of a complete binary toxin (actin-specific ADP-ribosyltransferase) by *Clostridium difficile* CD196.. Infect Immun.

[13] Popoff MR, Rubin EJ, Gill DM, Boquet P (1988). Actin-specific ADP-ribosyltransferase produced by a *Clostridium difficile* strain.. Infect Immun.

[14] Barth H, Aktories K, Popoff MR, Stiles BG (2004). Binary bacterial toxins: biochemistry, biology, and applications of common *Clostridium* and *Bacillus* proteins.. Microbiol Mol Biol Rev.

[15] Aktories K, Bärmann M, Ohishi I, Tsuyama S, Jakobs KH (1986). Botulinum C2 toxin ADP-ribosylates actin.. Nature.

[16] Vandekerckhove J, Schering B, Bärmann M, Aktories K (1987). *Clostridium perfringens* iota toxin ADP-ribosylates skeletal muscle actin in Arg-177.. FEBS Lett.

[17] Wegner A, Aktories K (1988). ADP-ribosylated actin caps the barbed ends of actin filaments.. J Biol Chem.

[18] Aktories K, Wegner A (1992). Mechanisms of the cytopathic action of actin-ADP-ribosylating toxins.. Mol Microbiol.

[19] Wiegers W, Just I, Müller H, Hellwig A, Traub P (1991). Alteration of the cytoskeleton of mammalian cells cultured in vitro by *Clostridium botulinum* C2 toxin and C3 ADP-ribosyltransferase.. Eur J Cell Biol.

[20] Gülke I, Pfeifer G, Liese J, Fritz M, Hofmann F (2001). Characterization of the enzymatic component of the ADP-ribosyltransferase toxin CDTa from *Clostridium difficile*.. Infect Immun.

[21] Coluccio LM, Bretscher A (1989). Reassociation of microvillar core proteins: making a microvillar core in vitro.. J Cell Biol.

[22] Fath KR, Burgess DR (1995). Microvillus assembly. Not actin alone.. Curr Biol.

[23] Chhabra ES, Higgs HN (2007). The many faces of actin: matching assembly factors with cellular structures.. Nat Cell Biol.

[24] Gundersen GG, Khawaja S, Bulinski JC (1987). Postpolymerization Detyrosination of a-Tubulin: A Mechanism for Subcellular Differentiation of Microtubules.. J Cell Biol.

[25] Palazzo A, Ackerman B, Gundersen GG (2003). Cell biology: Tubulin acetylation and cell motility.. Nature.

[26] Spector I, Shochet NR, Blasberger D, Kashman Y (1989). Latrunculins–novel marine macrolides that disrupt microfilament organization and affect cell growth: I. Comparison with cytochalasin D.. Cell Motil Cytoskeleton.

[27] Bubb MR, Senderowicz AMJ, Sausville EA, Duncan KLK, Korn ED (1994). Jasplakinolide, a cytotoxic natural product, induces actin polymerization and competitively inhibits the binding of phalloidin to F-actin.. J Biol Chem.

[28] Akhmanova A, Steinmetz MO (2008). Tracking the ends: a dynamic protein network controls the fate of microtubule tips.. Nat Rev Mol Cell Biol.

[29] Morrison EE, Wardleworth BN, Askham JM, Markham AF, Meredith DM (1998). EB1, a protein which interacts with the APC tumour suppressor, is associated with the microtubule cytoskeleton throughout the cell cycle.. Oncogene.

[30] Mimori-Kiyosue Y, Shiina N, Tsukita S (2000). The dynamic behavior of the APC-binding protein EB1 on the distal ends of microtubules.. Curr Biol.

[31] Coquelle FM, Caspi M, Cordelieres FP, Dompierre JP, Dujardin DL (2002). LIS1, CLIP-170's key to the dynein/dynactin pathway.. Mol Cell Biol.

[32] Hoogenraad CC, Akhmanova A, Grosveld F, De Zeeuw CI, Galjart N (2000). Functional analysis of CLIP-115 and its binding to microtubules.. J Cell Sci.

[33] Mimori-Kiyosue Y, Grigoriev I, Lansbergen G, Sasaki H, Matsui C (2005). CLASP1 and CLASP2 bind to EB1 and regulate microtubule plus-end dynamics at the cell cortex.. J Cell Biol.

[34] Akhmanova A, Hoogenraad CC, Drabek K, Stepanova T, Dortland B (2001). Clasps are CLIP-115 and -170 associating proteins involved in the regional regulation of microtubule dynamics in motile fibroblasts.. Cell.

[35] Kodama A, Karakesisoglou I, Wong E, Vaezi A, Fuchs E (2003). ACF7: an essential integrator of microtubule dynamics.. Cell.

[36] Carter GP, Lyras D, Allen DL, Mackin KE, Howarth PM (2007). Binary toxin production in *Clostridium difficile* is regulated by CdtR, a LytTR family response regulator.. J Bacteriol.

[37] Lawley TD, Clare S, Walker AW, Goulding D, Stabler RA (2009). Antibiotic treatment of *Clostridium difficile* carrier mice triggers a supershedder state, spore-mediated transmission, and severe disease in immunocompromised hosts.. Infect Immun.

[38] Chen X, Katchar K, Goldsmith JD, Nanthakumar N, Cheknis A (2008). A mouse model of *Clostridium difficile*-associated disease.. Gastroenterology.

[39] Sarma-Rupavtarm RB, Ge Z, Schauer DB, Fox JG, Polz MF (2004). Spatial distribution and stability of the eight microbial species of the altered schaedler flora in the mouse gastrointestinal tract.. Appl Environ Microbiol.

[40] Uematsu Y, Kogo Y, Ohishi I (2007). Disassembly of actin filaments by botulinum C2 toxin and actin-filament-disrupting agents induces assembly of microtubules in human leukaemia cell lines.. Biol Cell.

[41] Whipple RA, Cheung AM, Martin SS (2007). Detyrosinated microtubule protrusions in suspended mammary epithelial cells promote reattachment.. Exp Cell Res.

[42] Myers KA, He Y, Hasaka TP, Baas PW (2006). Microtubule transport in the axon: Re-thinking a potential role for the actin cytoskeleton.. Neuroscientist.

[43] Rogers SL, Rogers GC, Sharp DJ, Vale RD (2002). Drosophila EB1 is important for proper assembly, dynamics, and positioning of the mitotic spindle.. J Cell Biol.

[44] Tirnauer JS, O'Toole E, Berrueta L, Bierer BE, Pellman D (1999). Yeast Bim1p promotes the G1-specific dynamics of microtubules.. J Cell Biol.

[45] Lansbergen G, Akhmanova A (2006). Microtubule plus end: a hub of cellular activities.. Traffic.

[46] Wittmann T, Waterman-Storer CM (2005). Spatial regulation of CLASP affinity for microtubules by Rac1 and GSK3beta in migrating epithelial cells.. J Cell Biol.

[47] Etienne-Manneville S, Hall A (2002). Rho GTPases in cell biology.. Nature.

[48] Jaffe AB, Hall A (2005). Rho GTPases: biochemistry and biology.. Annu Rev Cell Dev Biol.

[49] Ridley AJ (2006). Rho GTPases and actin dynamics in membrane protrusions and vesicle trafficking.. Trends Cell Biol.

[50] Goode BL, Eck MJ (2007). Mechanism and function of formins in the control of actin assembly.. Annu Rev Biochem.

[51] Pollard TD (2007). Regulation of actin filament assembly by Arp2/3 complex and formins.. Annu Rev Biophys Biomol Struct.

[52] Rodriguez OC, Schaefer AW, Mandato CA, Forscher P, Bement WM (2003). Conserved microtubule-actin interactions in cell movement and morphogenesis.. Nat Cell Biol.

[53] Wen Y, Eng CH, Schmoranzer J, Cabrera-Poch N, Morris EJ (2004). EB1 and APC bind to mDia to stabilize microtubules downstream of Rho and promote cell migration.. Nat Cell Biol.

[54] Gundersen GG, Wen Y, Eng CH, Schmoranzer J, Cabrera-Poch N (2005). Regulation of microtubules by Rho GTPases in migrating cells.. Novartis Found Symp.

[55] Bartolini F, Moseley JB, Schmoranzer J, Cassimeris L, Goode BL (2008). The formin mDia2 stabilizes microtubules independently of its actin nucleation activity.. J Cell Biol.

[56] Watabe-Uchida M, John KA, Janas JA, Newey SE, van AL (2006). The Rac activator DOCK7 regulates neuronal polarity through local phosphorylation of stathmin/Op18.. Neuron.

[57] Cau J, Hall A (2005). Cdc42 controls the polarity of the actin and microtubule cytoskeletons through two distinct signal transduction pathways.. J Cell Sci.

[58] Fukata M, Watanabe T, Noritake J, Nakagawa M, Yamaga M (2002). Rac1 and Cdc42 capture microtubules through IQGAP1 and CLIP-170.. Cell.

[59] Ivanov AI, Hopkins AM, Brown GT, Gerner-Smidt K, Babbin BA (2008). Myosin II regulates the shape of three-dimensional intestinal epithelial cysts.. J Cell Sci.

[60] Geric B, Carman RJ, Rupnik M, Genheimer CW, Sambol SP (2006). Binary toxin-producing, large clostridial toxin-negative *Clostridium difficile* strains are enterotoxic but do not cause disease in hamsters.. J Infect Dis.

[61] Bartlett JG (2008). Historical perspectives on studies of *Clostridium difficile* and *C. difficile* infection.. Clin Infect Dis.

[62] Cerquetti M, Serafino A, Sebastianelli A, Mastrantonio P (2002). Binding of *Clostridium difficile* to Caco-2 epithelial cell line and to extracellular matrix proteins.. FEMS Immunol Med Microbiol.

[63] Calabi E, Calabi F, Phillips AD, Fairweather NF (2002). Binding of *Clostridium difficile* surface layer proteins to gastrointestinal tissues.. Infect Immun.

[64] Tasteyre A, Barc MC, Collignon A, Boureau H, Karjalainen T (2001). Role of FliC and FliD flagellar proteins of *Clostridium difficile* in adherence and gut colonization.. Infect Immun.

[65] Ohishi I, DasGupta BR, Eklund MW, Dowell VR (1987). Moleculare structure and biological activities of *Clostridium botulinum* C2 toxin.. Avian Botulism.

[66] Barth H, Blöcker D, Behlke J, Bergsma-Schutter W, Brisson A (2000). Cellular uptake of *Clostridium botulinum* C2 toxin requires oligomerization and acidification.. J Biol Chem.

[67] Blöcker D, Behlke J, Aktories K, Barth H (2001). Cellular uptake of the binary *Clostridium perfringens* iota-toxin.. Infect Immun.

[68] Barth H, Hofmann F, Olenik C, Just I, Aktories K (1998). The N-terminal part of the enzyme component (C2I) of the binary *Clostridium botulinum* C2 toxin interacts with the binding component C2II and functions as a carrier system for a Rho ADP-ribosylating C3-like fusion toxin.. Infect Immun.

[69] Just I, Selzer J, Hofmann F, Aktories K, Aktories K (1997). *Clostridium difficile* toxin B as a probe for Rho GTPases.. Bacterial toxins - Tools in cell biology and pharmacology.

[70] Wehland J, Willingham MC, Sandoval IV (1983). A rat monoclonal antibody reacting specifically with the tyrosylated form of alpha-tubulin. I. Biochemical characterization, effects on microtubule polymerization in vitro, and microtubule polymerization and organization in vivo.. J Cell Biol.

[71] Stepanova T, Slemmer J, Hoogenraad CC, Lansbergen G, Dortland B (2003). Visualization of microtubule growth in cultured neurons via the use of EB3-GFP (end-binding protein 3-green fluorescent protein).. J Neurosci.

