# Supplementary figures and images for: Clostridium difficile Toxin CDT Induces Formation of Microtubule-Based Protrusions and Increases Adherence of Bacteria
Source: PLoS Pathog. 2009 Oct 16;5(10):e1000626. doi: 10.1371/journal.ppat.1000626 (PMC2757728; doi:10.1371/journal.ppat.1000626)

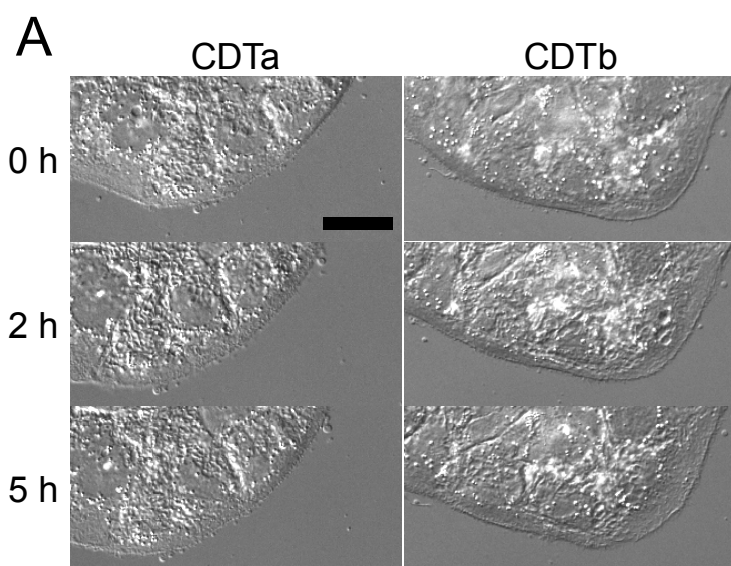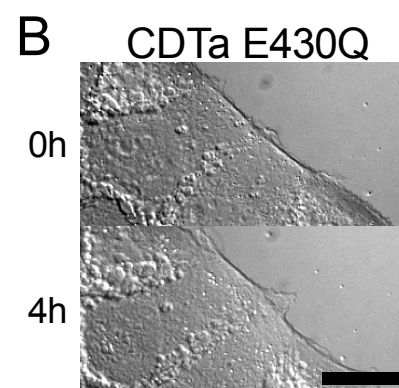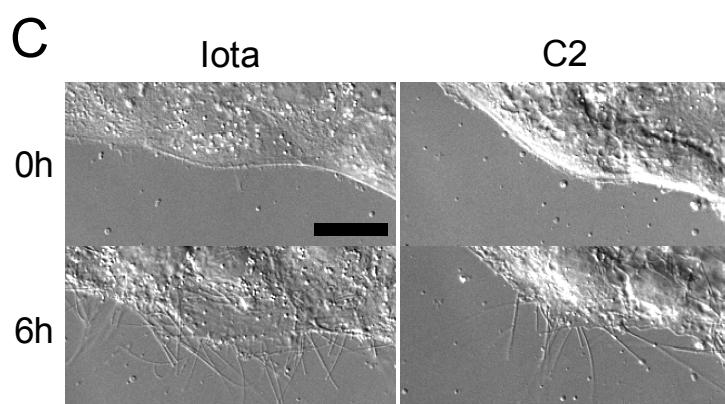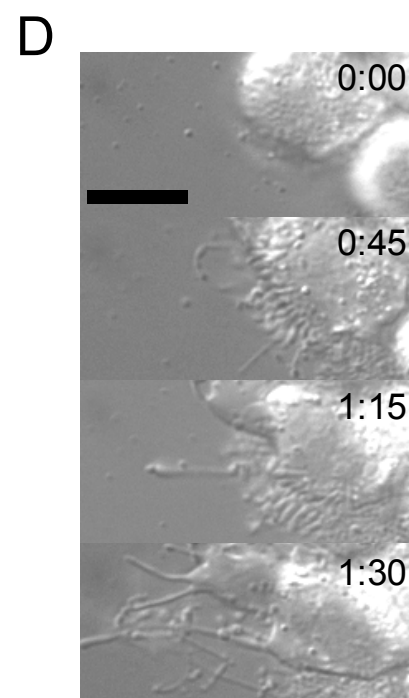

Supplement: Figure S1 — Specificity of toxin-induced formation of protrusions. (A) Both CDT components are necessary for protrusion formation. Series of DIC time-lapse images of Caco-2 cells treated with 20 ng/ml CDTa or 40 ng/ml CDTb. Subconfluent Caco-2 cells were treated with each toxin component for 0, 2 and 5 h. No formation of protrusions was observed when only one toxin component was added. Bar, 20 µm. (B) A catalytic inactive mutant of CDTa E430Q did not induce protrusion formation. DIC time-lapse microscopy of Caco-2 cells. Subconfluent Caco-2 cells were treated with 20 ng/ml CDTa E430Q and 40 ng/ml CDTb. No formation of protrusions was observed. (C) The ADP-ribosylating toxins iota toxin and C2 toxin induce formation of protrusions. DIC time-lapse microscopy of Caco-2 cells. Subconfluent Caco-2 cells were treated with 100 ng/ml iota toxin Ia (enzyme component) and 200 ng/ml iota toxin Ib (binding component) (iota, left panel) or with 250 ng/ml C2I and 500 ng/ml C2II (C2, right panel) for 6 h. Bar, 20 µm. (D) Primary colonocytes from the rat gut form protrusions after toxin treatment. DIC time-lapse microscopy of primary rat colonocytes after 1.5 days in vitro. Rat colonocytes were prepared as described in the Methods section and cultured for 1.5 days on a collagen-coated glass surface. The primary cells were treated with 20 ng/ml CDTa and 40 ng/ml CDTb. Pictures were taken after 0, 45, 75 and 90 min, respectively. In each panel the incubation time (h∶min) is indicated. Bar, 10 µm. (0.71 MB PDF) [file ppat.1000626.s001.pdf]

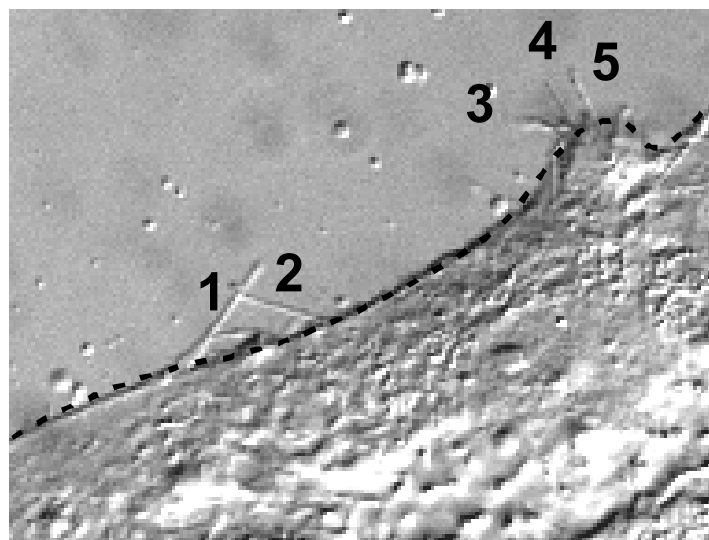

Supplement: Figure S2 — Quantification method of toxin-induced formation of protrusions. The lengths of protrusions (indicated 1–5) were determined by using the Metamorph software and the sum normalized by the respective section of cell perimeter (indicated as dashed line). Here Caco-2 cells were used and the toxin concentration was 20 ng/ml CDTa and 40 ng/ml CDTb. Incubation time was 1 h. (0.05 MB PDF) [file ppat.1000626.s002.pdf]

A

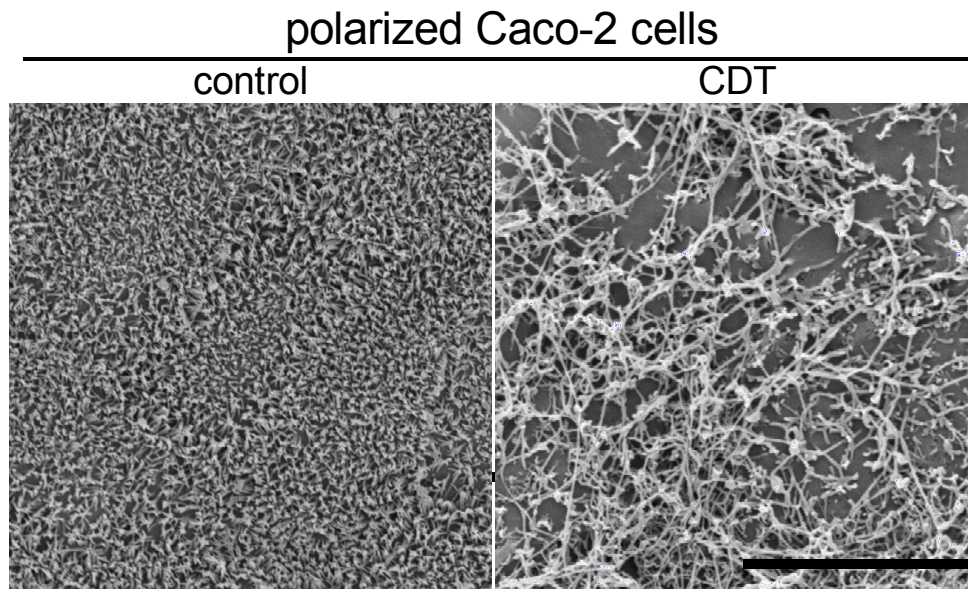

B

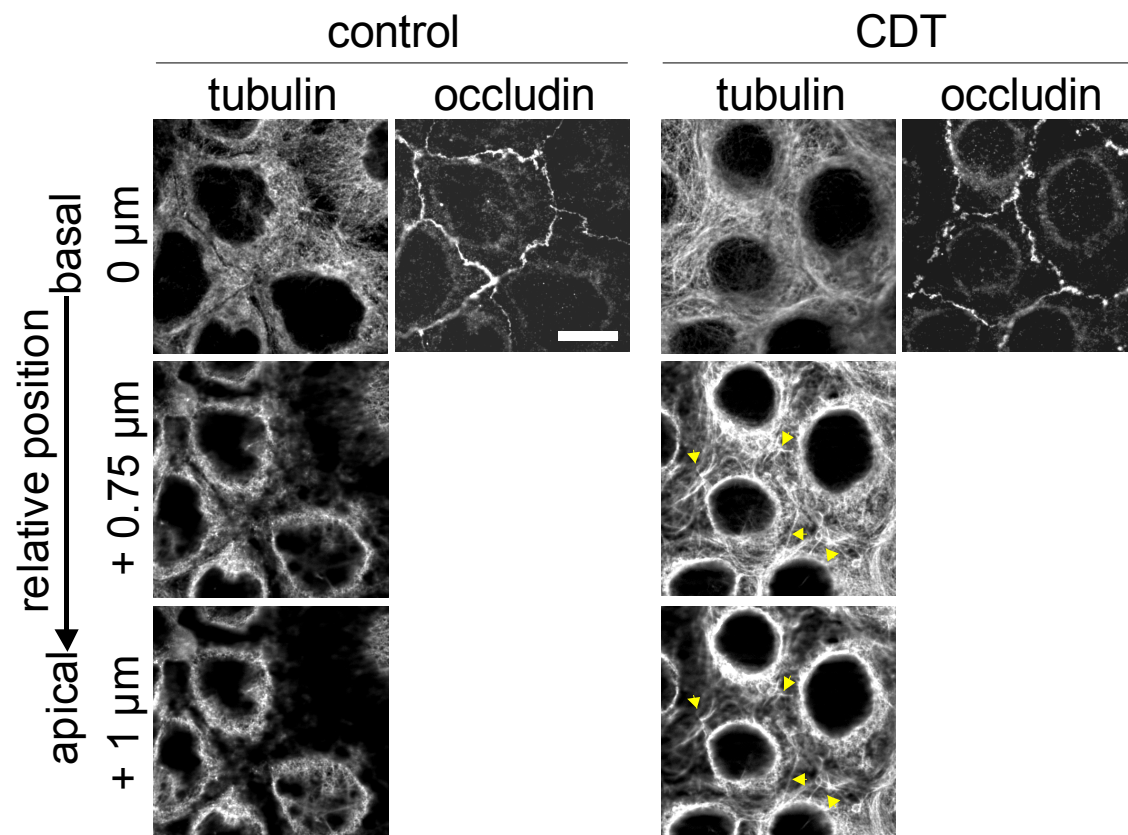

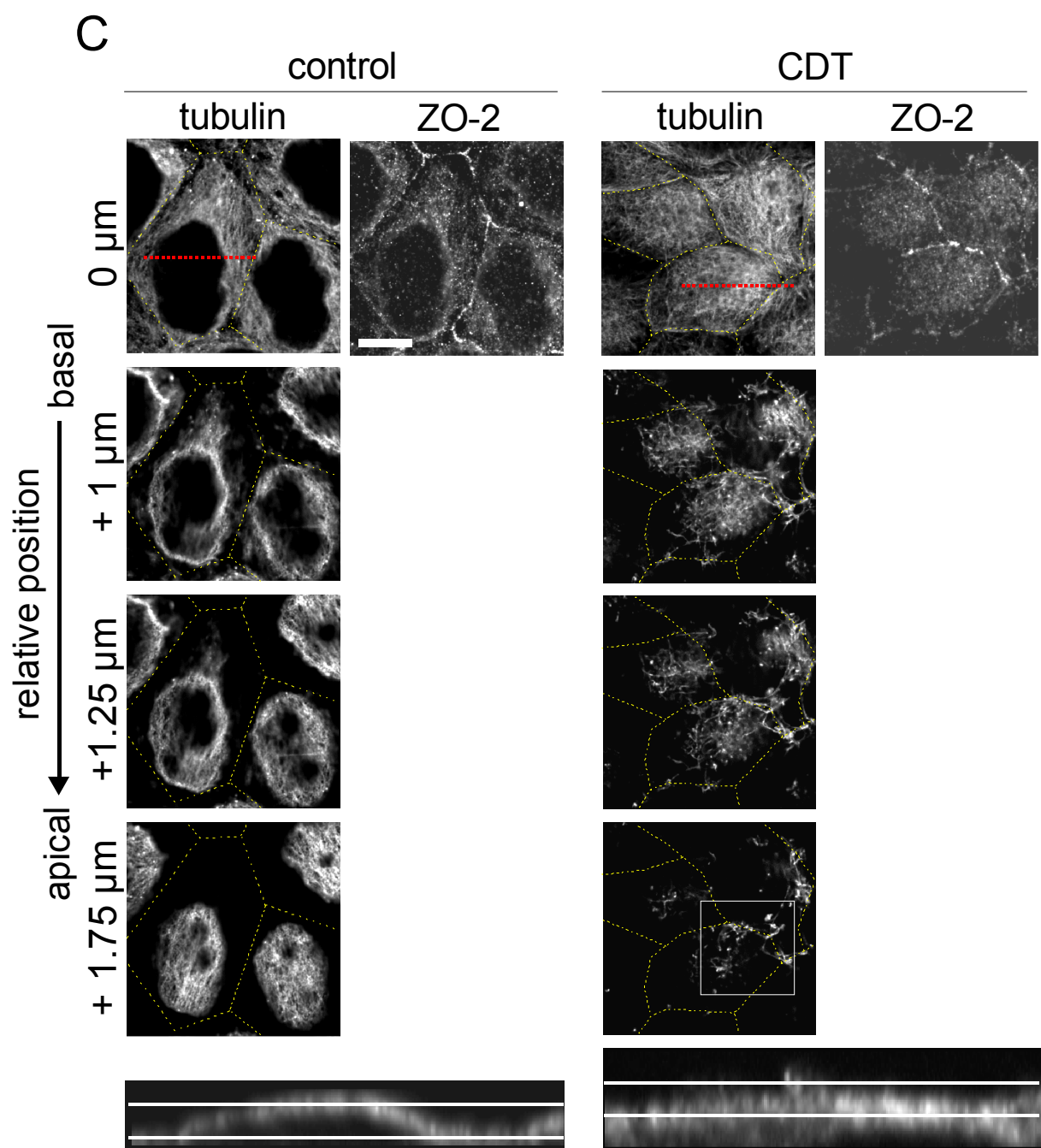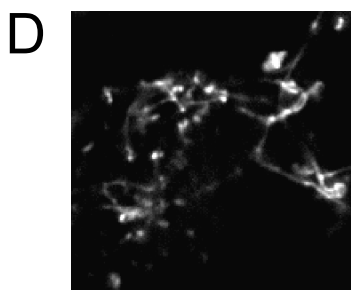

Supplement: Figure S3 — Visualization microtubule-based protrusions on polarized Caco-2 cells. (A) Caco-2 cells were grown on filters for 2 weeks to ensure polarization. Cells were treated with 20 ng/ml CDTa and 40 ng/ml CDTb. Untreated control cells show microvilli at the cell surface. On CDT-treated cells, microvilli disappeared and showed pronounced formation of protrusions. Scale bar represents 10 µm. (B) Confluent Caco-2 cells were grown for 1.5 weeks. The polarized cells were treated with 20 ng/ml CDTa and 40 ng/ml CDTb and fixed after 2 h or remained untreated as control cells. Confocal images of indirect immunofluorescence of α-tubulin and occludin were acquired as a Z-stack. Yellow arrows mark microtubule bundles at the cell surface. The relative position of the picture in the Z-stack is indicated on the left. Scale bar represents 10 µm. (C) Confluent Caco-2 cells grown for 1.5 weeks (as in S3B). The polarized cells were treated with 20 ng/ml CDTa and 40 ng/ml CDTb and fixed after 2 h or remained untreated as control cells. Confocal images of indirect immunofluorescence of α-tubulin and ZO-2 were acquired as a Z-stack. Yellow dashed lines represent cell borders delineated according to the ZO-2 staining. The relative position of the picture in the Z-stack is indicated on the left. The real position is shown in the cut view below. The cut view is reconstructed along the red line in the upper picture of the Z-stack. CDT treated cells are higher. As a result the area of the nucleus is not represented in the shown Z-stack of CDT-treatment. Scale bar represents 10 µm. (D) Magnification of the white square in Figure S3C (right panel), showing the microtubule-based protrusion meshwork at the cell surface. (3.07 MB PDF) [file ppat.1000626.s003.pdf]

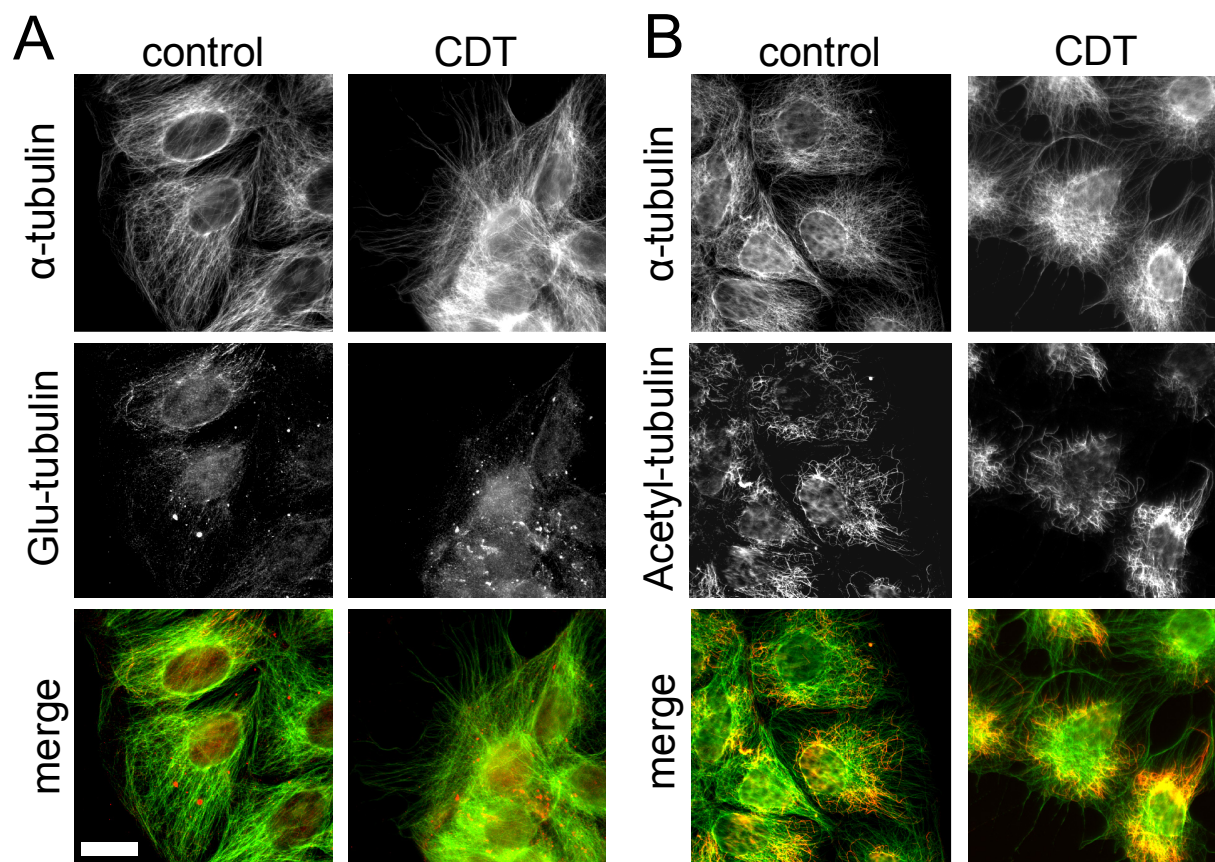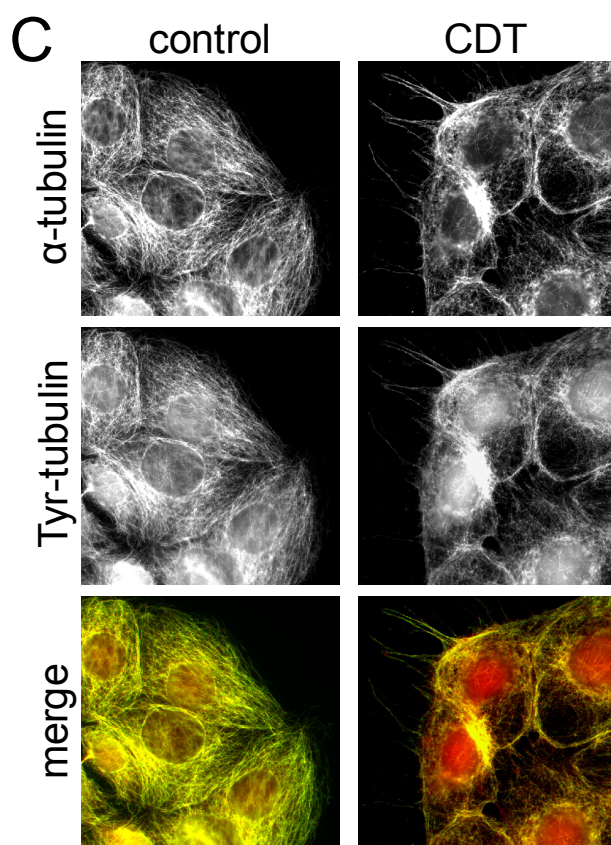

Supplement: Figure S4 — Staining of posttranslationally modified tubulin. Subconfluent Caco-2 cells were treated with 20 ng/ml CDTa and 40 ng/ml CDTb and fixed after 2 h or remained untreated as control cells. (A) Indirect immunofluorescence of α-tubulin (green) and detyrosinated tubulin (Glu-tubulin) (red). The amount of Glu-tubulin is not increased. Protrusions are not formed by Glu-tubulin. (B) Indirect immunofluorescence of α-tubulin (green) and acetylated tubulin (red). The amount of acetylated tubulin is not increased. Protrusions are not formed by actetylated tubulin. (C) Indirect immunofluorescence of α-tubulin (green) and tyrosinated tubulin (Tyr-tubulin) (red). The protrusions are formed by dynamic tyrosinated tubulin. Scale bar represents 20 μm (A,B,C). (2.19 MB PDF) [file ppat.1000626.s004.pdf]

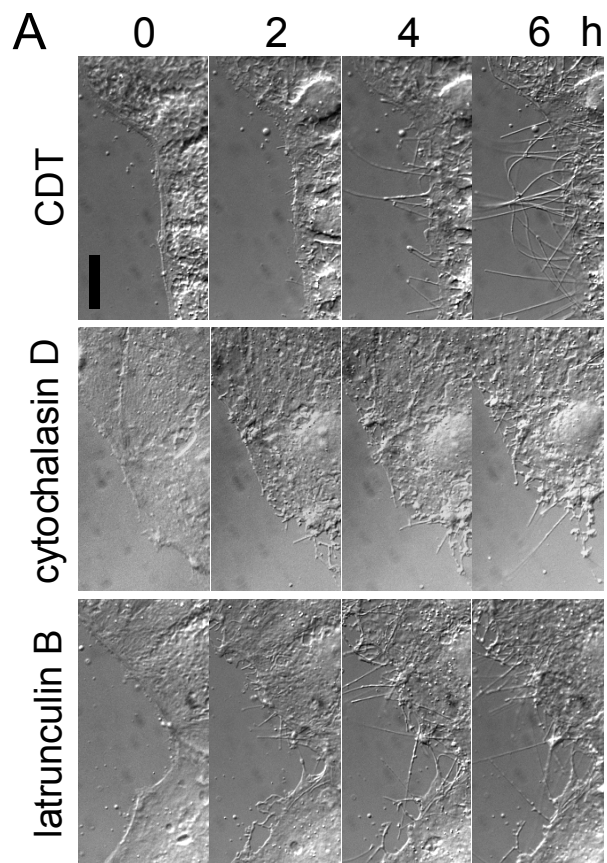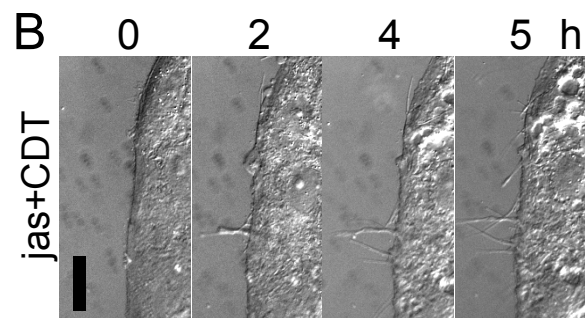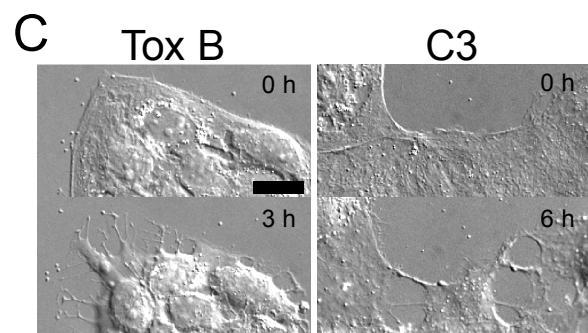

Supplement: Figure S5 — Influence of actin stabilizing and destabilizing drugs on the formation of microtubule-based protrusions. (A) Series of time-lapse images of cells treated with 20 ng/ml CDTa and 40 ng/ml CDTb, 5 µM latrunculin B and 1 µM cytochalasin D, respectively. Scale bar represents 20 µm. Actin destabilizing drugs can also induce protrusions, but less effective compared to CDT. (B) Series of time-lapse images of cells, which were treated with 500 nM jasplakinolide (jas) for 30 min. Then 20 ng/ml CDTa and 40 ng/ml CDTb were added and the formation of protrusions was monitored. Scale bar represents 20 µm. Actin stabilization can delay and decrease the formation of protrusions. (C) DIC time-lapse microscopy of Caco-2 cells. Subconfluent Caco-2 cells were treated with 300 ng/ml toxin B (Tox B) or 300 ng/ml C3 fusion toxin (C3) and 500 ng/ml C2II (to deliver the C3-fusion toxin into the cells) for the indicated times (h). The toxins were added to the cell culture medium. Incubation with toxins was continued until major morphological changes occurred. No formation of protrusions was observed under these conditions. Scale bar represents 20 µm. (0.87 MB PDF) [file ppat.1000626.s005.pdf]

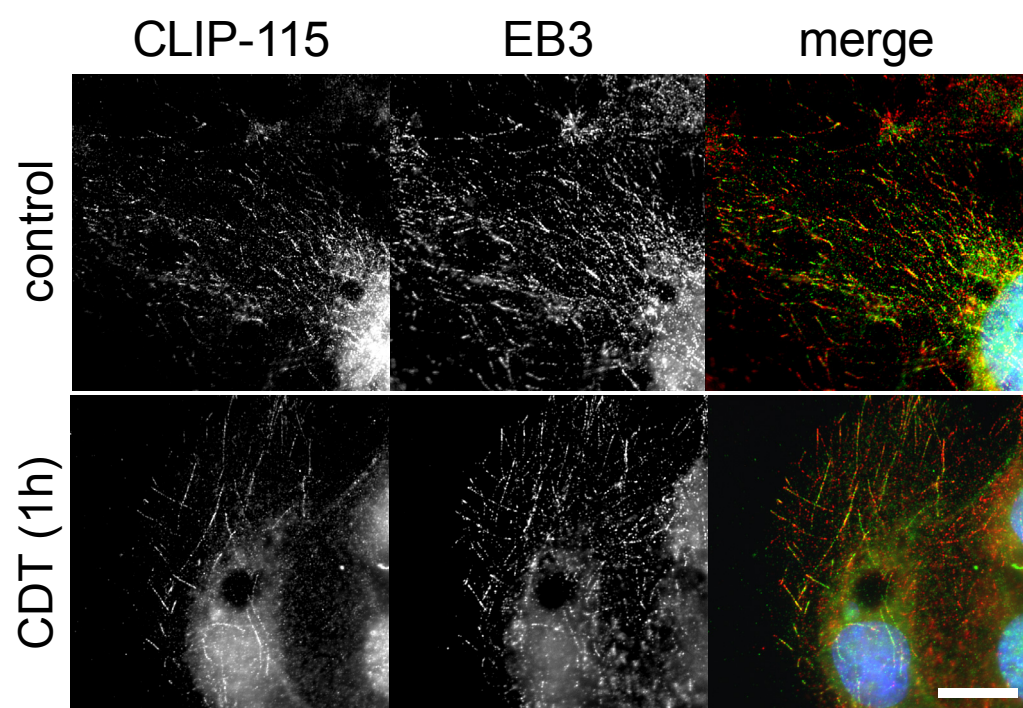

Supplement: Figure S6 — Influence of CDT on EB3 and CLIP-115 proteins. Subconfluent Caco-2 cells were treated with 20 ng/ml CDTa and 40 ng/ml CDTb and fixed after 1 h. Indirect immunofluorescence pictures of EB3 (red) by using anti-EB3 antibody and CLIP-115 (green) by using anti-CLIP-115 antibody in Caco-2 cells are shown. The nucleus was stained by DAPI (blue). Scale bar represents 20 µm. The increase of EB3 and CLIP-115 comet length after toxin treatment is seen. (0.84 MB PDF) [file ppat.1000626.s006.pdf]

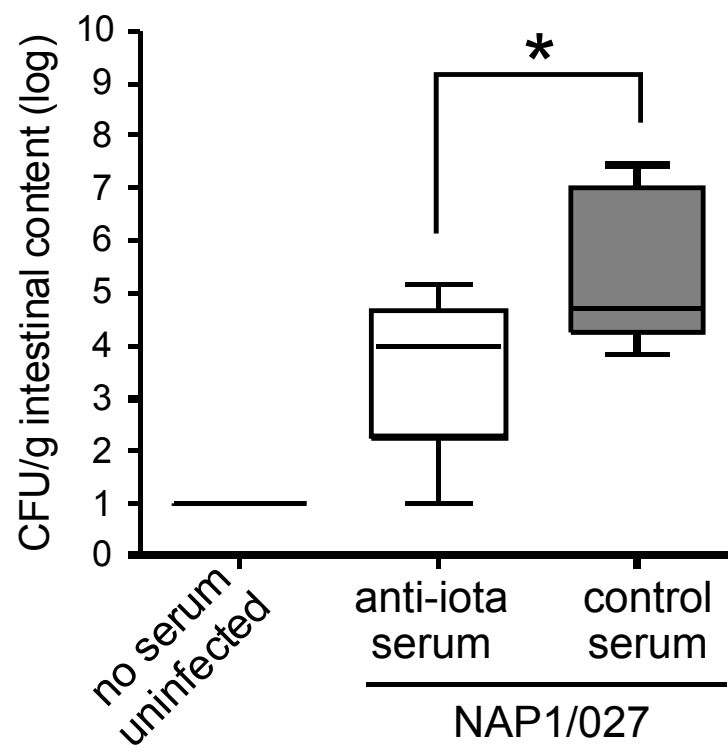

Supplement: Figure S7 — CDT increases bacterial loads of C. difficile in cecal content in vivo. Mice were infected by gavage with Nap1/027 (107 CFU) and subsequently treated with control serum or CDT-neutralizing anti-iota toxin serum. Cecal content was aseptically removed and homogenized. Bacterial loads in the cecal content from uninfected untreated mice, Nap1/027 (107 CFU) infected CDT-neutralizing anti-iota toxin serum treated mice and Nap1/027 infected control antiserum treated mice were determined by plating on C. difficile selective agar plates at different dilutions. The minimal detectable value was 10 CFU/g. The experiments were performed on 2 independent occasions with a total of 10 Nap1/027 infected mice treated with anti-iota CDT-neutralizing serum, 11 Nap1/027 infected mice treated with control serum and 4 control mice. Boxes indicate 25th and 75th percentiles, black bars indicate medians, and whiskers indicate data ranges. Y-axis is scaled logarithmically (log10). * indicates p≤0.05. (0.03 MB PDF) [file ppat.1000626.s007.pdf]

A

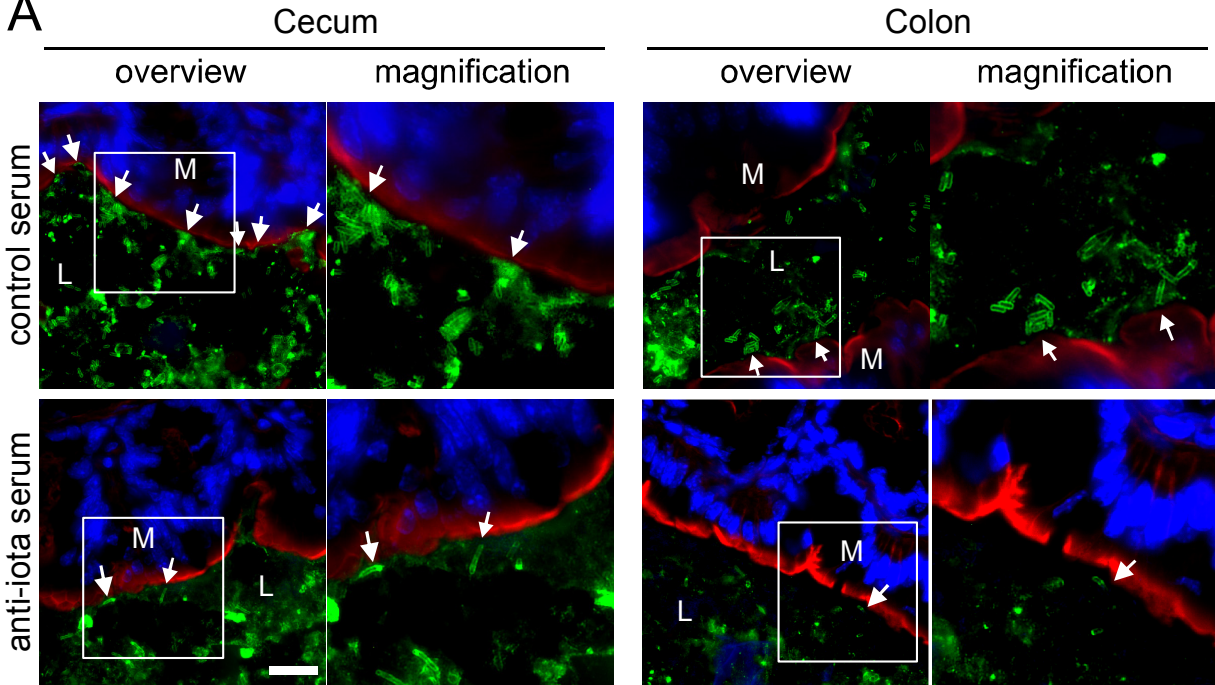

B

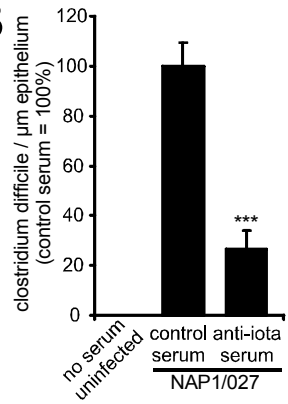

C

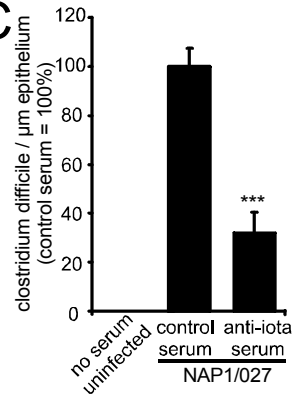

Supplement: Figure S8 — CDT increases adherence of C. difficile in the cecum and colon in vivo. (A) Mice were infected with Nap1/027 (107 CFU) and subsequently treated with control serum or CDT-neutralizing anti-iota toxin serum. Cryosections (7 µm) of PFA-fixed and cryo-embedded tissue from cecum and colon were immunostained for C. difficile. Actin was stained by TRITC-phalloidin and the nuclei by DAPI. M marks the mucosa and L marks the lumen of the intestine. White boxes are magnified. White arrows mark bacteria or bacterial aggregations adjacent to the epithelium. Calibration bar represents 20 µm. (B) Quantification of C. difficile directly adjacent to the epithelium in the cecum. For bacterial quantification, bacteria adjacent to the epithelium, stained with anti-C. difficile serum, within 3 µm of the epithelium were counted. If bacterial aggregations were attached to the epithelium all bacterial cells in the aggregation were counted. This was done for ≥15 optical fields per group. The number of C. difficile per µm mucosal surface was determined. Nap1/027 infected mice treated with control serum were set 100%. (C) Quantification of C. difficile directly adjacent to the epithelium in the colon. The number of C. difficile per µm mucosa was determined as in B. (0.85 MB PDF) [file ppat.1000626.s008.pdf]
